# WebView-Based Hybrid Analysis of Link and Event for On-Device QR Phishing Detection Framework

**DOI:** 10.3390/s26144412

**Published:** 2026-07-11

**Authors:** Jian Woo, Seungmin Lee, Inseok Park, Sejong Lee

**Affiliations:** 1 Department of Computer Engineering, Yeungnam University, 280 Daehak-ro, Gyeongsan 38541, Gyeongbuk, Republic of Korea; wkb6325@yu.ac.kr (J.W.); 22313529@yu.ac.kr (S.L.); ibsq123@yu.ac.kr (I.P.); 2 Department of Computer Science and Engineering, Yeungnam University, 280 Daehak-ro, Gyeongsan 38541, Gyeongbuk, Republic of Korea

**Keywords:** quishing detection, mobile security, dynamic analysis, static analysis

## Abstract

Quishing, a form of phishing conducted through QR codes, has emerged as a critical threat to user information security in mobile environments. Quishing attacks exploit the QR scanning workflow by opening malicious URLs in WebView and impersonating legitimate services to steal user credentials. Recent variants further evade static inspection by exposing credential-harvesting behavior only after user interaction, form submission, redirection, or page-state changes. In this paper, we propose WebView-Based Hybrid Analysis of Link and Event for On-Device QR Phishing Detection (WHALE), an On-Device multi-stage phishing detection framework based on an isolated *Sandbox WebView*. WHALE first loads the QR-decoded URL into the *Sandbox WebView* instead of directly delivering it to the *User WebView*, thereby separating the analysis process from the user session. In the static stage, WHALE extracts 54 features from the URL string, initial HTML, and DOM snapshot, and computes a static phishing risk score using a lightweight model. Inputs with uncertain static scores are forwarded to the dynamic stage. In the dynamic stage, WHALE inserts decoy credentials instead of real user credentials, triggers a controlled submit event, and analyzes 59 credential-flow state-transition features extracted before and after submission. The static model achieved an accuracy of 93.86%, precision of 93.08%, recall of 94.78%, and F1-score of 93.92%. The dynamic model achieved an accuracy of 0.915, precision of 0.895, recall of 0.946, specificity of 0.882, and F1-score of 0.920 on a source-group-disjoint independent test set. Real-device evaluation on a Samsung Galaxy S23 Ultra showed that WHALE maintains practical mobile overhead, with an average static internal runtime of 58.75 ms, dynamic internal runtime of 4165.42 ms, combined model inference time of 0.088 ms, and model asset size of 0.681 MB. These results demonstrate that WHALE can detect QR-based phishing threats On-Device while reducing user credential exposure through sandboxed credential-flow analysis.

## 1. Introduction

The Quick Response (QR) code is a type of barcode. QR codes serve as an efficient medium for delivering web links and enable immediate connection through scanning [1,2,3]. QR codes are used in a wide range of services, including payment, access control, and public information. Users can access webpages linked to QR codes through a camera scan without an additional input process [4,5,6,7]. However, QR codes can be abused because of inherent structural security vulnerabilities [8,9,10]. A representative cybercrime case uses QR codes containing malicious URLs to direct users to phishing sites for malicious purposes, such as extorting money or stealing user information [11,12]. A QR-based phishing attack is referred to as quishing [13]. Quishing exploits the basic QR code mechanism, where scanning renders a webpage in a WebView or launches a related application. Therefore, address visibility is often limited during execution. Even when the address is displayed, users still have difficulty determining whether the domain is malicious [14]. Moreover, repeated redirections rapidly change the domain, making continuous verification difficult [15]. The operating mechanism of QR codes structurally favors attackers and creates serious security vulnerabilities for users [16,17]. Recent quishing is increasingly difficult to identify using only static features such as URL strings and HTML structures [18]. In some cases, the page appears legitimate during the initial loading stage. Sensitive data transmission or additional redirection is triggered only after user interactions such as input and clicks [19,20,21,22]. Addressing the threats posed by evolving quishing requires comprehensive evaluation criteria that consider runtime behavior and user interactions [23,24]. Current quishing detection relies on server database queries based on static features [25,26]. Server-based detection approaches have the advantage of accurately blocking reported malicious domains [27]. However, the performance of conventional server-based detection is highly susceptible to latency in unstable network environments. Mobile environments with limited resources make these server-based methods inefficient in terms of latency and availability. Moreover, privacy threats from third parties must be considered, as sensitive user information could be exposed during the query process. Therefore, real-time quishing detection in mobile environments requires an On-Device approach that performs local analysis rather than relying on conventional server-side queries [28,29].

In this paper, we propose WebView-based Hybrid Analysis of Link and Event (WHALE), an On-Device approach to QR phishing detection. Unlike prior QR phishing scanners that mainly rely on URL-level static features, blacklist queries, or QR image patterns, WHALE focuses on the mobile execution path after QR scanning. The main novelty of WHALE is a staged On-Device architecture in which the scanned URL is first executed in an isolated *Sandbox WebView*, separated from the *User WebView*. WHALE then applies lightweight static analysis and invokes dynamic observation only when the static score is inconclusive. This design differs from conventional URL phishing detection and generic WebView sandboxing because it combines QR-triggered execution isolation, selective dynamic triggering, and runtime behavior observation within the mobile device. The proposed framework performs context-aware static and dynamic analysis by considering the specific characteristics of quishing. The proposed method preemptively loads the URL obtained from the QR scan in a *Sandbox WebView*, which is isolated from the user’s WebView. From the loaded page, the system extracts static features comprising the URL string and the initial HTML structure. A lightweight deep learning model then performs static analysis using these features as input. If the static analysis yields a conclusive result, the system either blocks the URL or releases it according to the static decision. For inputs whose static evidence remains insufficient, WHALE executes dynamic analysis inside the isolated *Sandbox WebView*. During dynamic analysis, WHALE does not rely on redirection chains or domain transitions as standalone evidence. Instead, it performs controlled decoy credential submission and observes credential-flow state transitions, including form-structure changes, credential-role transitions, hidden-input mutations, response signals, handler mutations, and credential-consumption behavior. Through this selective static and dynamic analysis approach, WHALE protects users against evolving QR phishing techniques while reducing the risk of exposing real user credentials to suspicious pages. The main contributions of this paper are as follows.

- **User-Centric On-Device Detection Model**
  The proposed framework is designed to perform risk assessment and dynamic execution monitoring after QR scanning entirely On-Device. Our approach incorporates a lightweight TensorFlow Lite model to perform autonomous quishing detection directly within the mobile environment. By eliminating dependency on a central server, the On-Device detection model minimizes latency and privacy risks while providing real-time protection for users.

- **QR Phishing Detection Architecture for Mobile Environments**
  We propose WHALE, a multi-stage QR phishing detection architecture optimized for mobile environments that integrates static and event-based dynamic analysis. Immediately following a QR scan, WHALE calculates risk using the URL and initial static features. If these static results are uncertain, dynamic analysis is triggered. Our selective approach optimally balances real-time performance with comprehensive detection coverage.

- ***Sandbox WebView*** **Framework Isolated from User Sessions**
  A dedicated *Sandbox WebView* is introduced to prevent user data leakage and mitigate adverse impacts during quishing detection. While conventional methods immediately execute QR-loaded URLs in the primary WebView, the proposed framework preemptively confines suspicious pages within an isolated virtual environment. Operating within this secure perimeter allows WHALE to safely observe credential-flow state transitions, including form-structure changes, credential-role transitions, hidden-input mutations, response signals, handler mutations, and credential-consumption behavior. Isolating these threats effectively shields the active user session from direct compromise by credential-harvesting attempts or malicious handlers.

## 2. Related Work

This section summarizes relevant studies for phishing detection during mobile QR scanning. QR phishing detection methodologies are primarily categorized into scanner-based, reference-based, and QR image-based approaches. Representative recent studies for each detection strategy are introduced below. Recent studies dedicated to quishing detection are presented in Table 1.

Rafsanjani et al. proposed QsecR, a detection pipeline that integrates malicious URL identification directly into Android QR scanners [30]. QsecR computes risk scores through the combination of static features extracted immediately after scanning. These features include lexical signals derived from the URL string, host-based attributes, and page metadata. The scanner analyzes the URL before user access, augmenting detection evidence through external reputation data such as blacklists. Maliciousness is then determined via a learning-based classifier using the URL string-based features, host-based attributes, and page metadata. Through these approaches, the architecture ensures rapid decision-making, effectively meeting critical real-time demands within mobile environments. However, QsecR relies only on initial static features, making it less effective against redirection chains and login forms triggered by user events.

Liu et al. address missed detections in reference-based phishing identification resulting from static reference lists [31]. Reference-based detection utilizes pre-acquired data from legitimate brands or services to evaluate suspicious pages. A suspicious page is classified as phishing if it exhibits high visual similarity to the reference data despite low domain consistency. However, this approach makes it difficult to detect targets absent from the reference list, such as new brands or localized services. Moreover, visual similarity alone proves insufficient for distinguishing credential-harvesting pages that lack clear brand cues.

**Table 1:** Summary of related work on QR-code-based phishing and malicious QR code detection.

Year	Title	Authors	Description
2021	Quick Response Code Validation and Phishing Detection Tool [32]	Ismail et al.	Performs URL validation and phishing detection at the scanner stage after QR scanning. Provides rapid warnings based on URL-level static cues and rule-based analysis. Observation of final behavior-based cues, such as redirection and post-event changes, is limited.
2023	QsecR: Secure QR Code Scanner According to a Novel Malicious URL Detection Framework [30]	Rafsanjani et al.	Integrates a malicious URL detection pipeline into an Android QR scanner. Combines blacklist, URL lexical, host-based, and limited content-based static features. Its static-signal-centered design has limitations in handling conditional exposure and multi-step redirection.
2023	Knowledge Expansion and Counterfactual Interaction for Reference-Based Phishing Detection [31]	Liu et al.	Addresses the fixed-list limitation of reference-based phishing detection through runtime reference expansion. Combines legitimacy verification of added references with interaction-based behavioral signal observation. Interaction and verification procedures increase latency and resource consumption.
2024	QR Shield: A Dual Machine Learning Approach Towards Securing QR Codes [33]	Almousa et al.	Proposes a dual machine learning-based detection procedure for QR-related threats. Constructs classification models mainly using static features of URLs embedded in QR codes. Its static-feature-centered design still requires separate verification of dynamic behavior on the final landing page.
2024	Detection of QR Code-based Cyberattacks Using a Lightweight Deep Learning Model [34]	Sarkhi et al.	Presents a lightweight deep learning approach for classifying QR code-based attacks. Uses QR code images as input and demonstrates the feasibility of on-device deployment. Signals revealed during web execution, such as redirection and post-event changes, are difficult to handle directly.
2025	Detecting Quishing Attacks with Machine Learning Techniques Through QR Code Analysis [8]	Trad et al.	Detects maliciousness by analyzing the structure and pixel patterns of QR codes using machine learning. Relies on QR image analysis without requiring URL information. Image-based classification alone is insufficient to replace verification of phishing intent based on final web behavior.
2025	Efficient Malicious QR Code Detection System Using an Advanced Deep Learning Approach [35]	Alsulami et al.	Determines maliciousness through QR image-based feature extraction and classification. Shows that QR image analysis can be used for efficient detection. QR image-based detection makes it difficult to collect features that reflect interaction processes on the landing page.

The method in [36] mitigates the limitations of the reference list through active expansion during execution. When brand cues are insufficient to reveal unauthorized credential acquisition intent through static analysis, the system collects behavior-based features. Adaptive detection enables robust phishing identification even for inputs with limited static evidence, expanding the overall coverage. This approach improves the detection method through a legitimacy verification step that prevents false positives caused by indiscriminate reference expansion. However, combining reference expansion, verification, and interaction execution incurs significant latency and high computational overhead. The real-time requirements of mobile settings are fundamentally at odds with the high-latency nature of these traditional detection methods.

Trad et al. proposed a QR image-based detection approach that utilizes machine learning to identify maliciousness through the structural and pixel patterns of QR codes [8]. The study in [8] analyzes the visual structure of QR codes to detect phishing attempts without relying on URL information. This allows the approach to function as a pre-filter prior to the webpage loading phase. However, QR image-based classification remains insufficient for detecting behavior-based phishing that manifests through interactions after actual webpage loading. Scanner and QR image-based detection methods provide rapid processing, but find it difficult to identify dynamic threats such as redirections and user events. Reference-based approaches often suffer from high computational overhead, rendering them unsuitable for On-Device environments. Therefore, this paper proposes a hybrid detection framework that integrates static and dynamic analysis to address the limitations of existing detection methods. Compared with scanner-based and QR image-based methods, WHALE does not stop at pre-loading static inspection but observes the execution behavior of the decoded URL in an isolated mobile WebView. Compared with conventional URL phishing classifiers, WHALE is designed for the QR scanning workflow, where the URL is analyzed before being delivered to the user session. Compared with general sandboxing approaches, WHALE selectively combines static scoring and event-based dynamic observation to support real-time On-Device QR phishing detection. Within this framework, the static stage calculates risk using initial static features. When it is difficult for static analysis to yield a definitive result, the system executes a behavior-based dynamic detection process. This approach ensures the effective utilization of limited On-Device resources while responding comprehensively to diverse quishing threats.

## 3. Methods

This section describes WHALE, a proposed hybrid phishing detection framework that incorporates static and dynamic analysis. The static stage is then detailed, focusing on quantifying risk levels using features extracted from the input URL. Furthermore, the behavior-based dynamic detection process is explained, highlighting its execution for cases where static analysis yields ambiguous results. Symbols used in this section follow the notation presented in Table 2.

### 3.1. Threat Model

The threat model considered in this paper assumes a scenario in which a user interacts with a QR code via a mobile device. The QR code qr captured through the device’s camera is processed by the Google ML Kit Barcode Scanning API (Google LLC., Mountain View, CA, USA) to decode the URL *u* [37]. The decoding procedure is formally defined by Equation (1).

(1) u←QRDecode(qr)

The adversary A distributes QR codes embedded with phishing URLs to target unspecified users. The phishing URL *u* is assumed to mimic a legitimate service to enhance the success of the deception. With g(·) defined as the phishing detection rate, A attempts to minimize g(u) to bypass detection mechanisms. The goal of A is to compromise user privacy, which is defined by Equation (2). In addition to this general QR phishing scenario, we further define the adversarial capabilities considered in the dynamic analysis stage. The adversary may operate a QR-triggered credential-harvesting page that avoids immediate redirection, remains on the same visible URL, modifies form handlers, mutates hidden inputs, exposes credential requests only after user interaction, or changes the credential form after submission. Therefore, the dynamic stage is not modeled as a simple redirection detector. Instead, WHALE evaluates whether credential-flow behavior is suspicious before real user credentials are released.

(2) $$\arg\min_{u}g\left(u\right)$$

Our goal is to identify and intercept phishing URLs in QR codes to ensure user privacy. The detection result $\hat{y}\left(u\right)$ classifies *u* as phishing as it approaches 1, and legitimate as it approaches 0. With $U_{\mathrm{phishing}}$ and $U_{\mathrm{legitimate}}$ defined as each set, the system maximizes the detection probability for $U_{\mathrm{phishing}}$. Maximizing this detection probability protects user privacy, as defined in Equation (3).

(3) $$\begin{array}{cc} \arg\max_{\hat{y}} & \left(\mathrm{Pr}[\hat{y}\left(u\right)=1|u\in U_{\mathrm{phishing}}\right]\\ & \;-\mathrm{Pr}[\hat{y}\left(u\right)=1|u\in U_{\mathrm{legitimate}}]) \end{array}$$

The first term in Equation (3), $\mathrm{Pr}[\hat{y}\left(u\right)=1|u\in U_{\mathrm{phishing}}]$, represents the probability of correctly identifying a phishing URL. The second term, $\mathrm{Pr}[\hat{y}\left(u\right)=1|u\in U_{\mathrm{legitimate}}]$, denotes the probability of misclassifying a legitimate URL as phishing. An increase in $\mathrm{Pr}[\hat{y}\left(u\right)=1|u\in U_{\mathrm{legitimate}}]$ directly leads to a higher false positive rate. Therefore, the proposed method is designed to maximize overall detection performance by increasing phishing detection accuracy while minimizing the false positive rate. The dynamic stage is invoked only when the static analysis yields an ambiguous result and the *Sandbox WebView* reaches a credential-relevant page or a credential-request page (CRP) candidate. In this stage, WHALE does not release real user credentials to the page. Instead, it injects decoy credentials within the isolated *Sandbox WebView* and observes pre and post-submit credential-flow state transitions. The observed signals include credential-role changes, form mutation, hidden-input mutation, validation or retry signals, and credential-consumption behavior. Pages without observable decoy credential submission, non-credential QR scams, broken phishing kits, and CAPTCHA and MFA-gated flows are treated as outside the main binary dynamic evaluation and are discussed as limitations.

### 3.2. Overview

**Figure 1:**
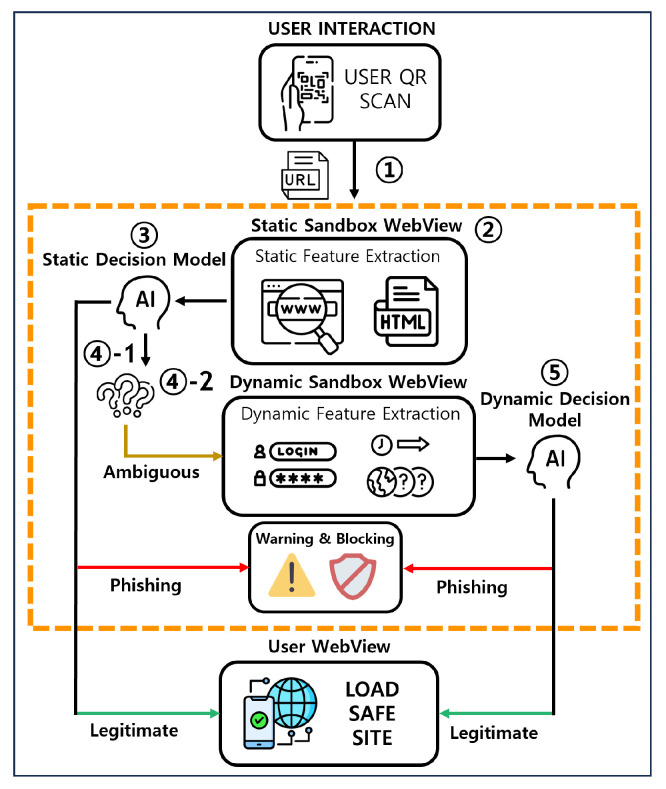
Overview of the proposed WebView-based hybrid analysis of link and event for the On-Device QR phishing detection framework.

Figure 1 illustrates the overall phishing analysis and detection workflow of WHALE. After a QR scan, the URL *u* is extracted according to the procedure defined in Equation (1). WHALE does not directly deliver the extracted URL *u* to the *User WebView*. Instead, the URL is first loaded into an isolated *Sandbox WebView*. This structure separates the detection process from the user session and prevents the scanned page from directly accessing user-side cookies, storage, cache, autofill data, and login state during analysis. The state isolation between the *Sandbox WebView* and the *User WebView* is expressed in Equation (4).

(4) $$\mathrm{State}\left(WV_{\mathrm{sandbox}}\right)\cap \mathrm{State}\left(WV_{\mathrm{user}}\right)=⌀$$

The components of State(·) include cookies, session information, storage, cache, autofill data, and login state. Therefore, even when a malicious page is loaded during analysis, the information accessible to the page remains confined to the sandbox context. WHALE follows a staged detection workflow. In the first stage, static analysis is performed using features extracted from the QR-decoded URL *u* and the initially loaded page content.

The static model computes a phishing risk score and applies a dual-threshold decision policy. Low-risk inputs are classified as safe, high-risk inputs are blocked, and uncertain inputs are forwarded to the dynamic detection stage. Thus, the dynamic analysis is not executed for every scanned URL. It is selectively invoked only when the static evidence is insufficient for a confident decision. For uncertain inputs, WHALE performs behavior-based dynamic analysis inside the *Sandbox WebView*. The dynamic stage targets credential-relevant pages or CRP candidates reached during sandbox execution. In this stage, WHALE does not insert real user credentials into the page. Instead, it inserts decoy credentials into detected credential fields, triggers a controlled submit event, and observes the page states before and after submission.

The dynamic model then evaluates the risk of the credential flow using state-transition features, including form-structure changes, credential-role transitions, hidden-input mutations, response signals, handler mutations, and credential-consumption behavior. This design enables WHALE to analyze suspicious credential-flow behavior without exposing real user credentials. A phishing decision leads WHALE to block access and present a warning to the user, as shown in the Warning & Blocking stage in Figure 1. A safe decision releases the verified URL to the *User WebView*, allowing the user to interact with the page. Algorithm 1 summarizes the operational procedure of the proposed WHALE framework. The detailed procedures of the static and dynamic stages are described in the *Static Phishing Detection* and *Dynamic Phishing Detection* sections, respectively.
**Algorithm 1** WHALE Detection Algorithm**Require:** *u* decoded from the QR scanner **Ensure:** Decision d∈{ALLOW,BLOCK}
$WV_{\mathrm{sandbox}}\leftarrow \mathrm{InitSandboxWebView}\left(u\right)$$h_{0}\leftarrow \mathrm{GetHTML}\left(WV_{\mathrm{sandbox}}\right)$$S_{\mathrm{init}}\leftarrow \left(u,h_{0}\right)$$E\leftarrow \{e_{1},e_{2},\ldots ,e_{k}\}$$x_{\mathrm{html}}\leftarrow \phi_{\mathrm{html}}\left(h_{0}\right)$$x_{\mathrm{url}}\leftarrow \phi_{\mathrm{url}}\left(u\right)$$x_{\mathrm{static}}\leftarrow \left[x_{\mathrm{html}}\;∥\;x_{\mathrm{url}}\right]$$\theta_{\mathrm{static}}\leftarrow f_{\mathrm{static}}\left(x_{\mathrm{static}}\right)$**if** $\theta_{\mathrm{static}}\geq T_{\mathrm{block}}$ **then**       **return** BLOCK**else if** $\theta_{\mathrm{static}}\leq T_{\mathrm{allow}}$ **then**       **return** ALLOW**else**       $x_{\mathrm{dynamic}}\leftarrow \Phi \left({\mathrm{State}}_{\mathrm{pre}},{\mathrm{State}}_{\mathrm{post}}\right)$       $\theta_{\mathrm{dynamic}}\leftarrow f_{\mathrm{dynamic}}\left(x_{\mathrm{dynamic}}\right)$       **if** $\theta_{\mathrm{dynamic}}\geq \tau_{\mathrm{dynamic}}$ **then**             **return** BLOCK       **else**             **return** ALLOW       **end if****end if**


### 3.3. Static Phishing Detection

**Figure 2:**
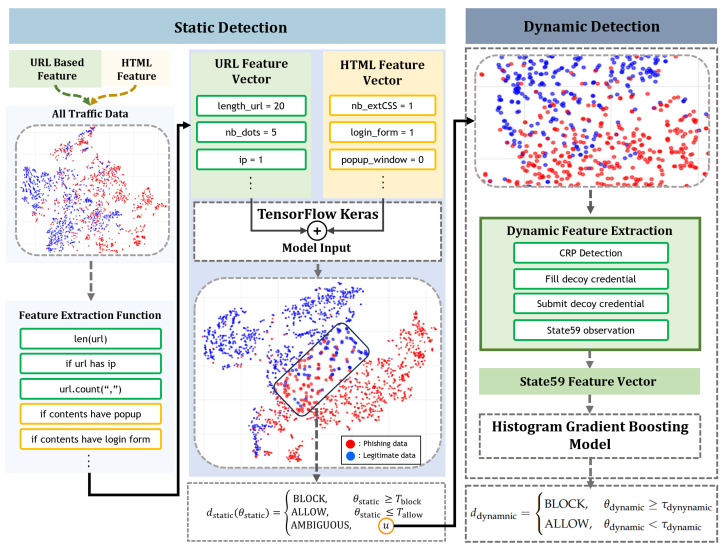
Static and dynamic detection process. The arrows indicate the processing flow. Red dots represent phishing data, and blue dots represent legitimate data.

The static stage calculates a risk score based on features acquired from the input *u* and HTML. Figure 2 illustrates the analytical process using extracted static features. WHALE loads *u* into the initialized $WV_{\mathrm{sandbox}}$ for static evaluation. Once loading is complete, the system collects the *u* string and an HTML snapshot representing the initial state of the page. The URL string *u* provides various extractable features. However, quishing attackers often employ URL shortening or complex obfuscation. Adversarial tactics of such nature reduce the reliability of detection when relying solely on the URL string. WHALE addresses such limitations through the integration of initial HTML features. Initial HTML snapshots provide crucial indicators like input fields and form structures. Consequently, the static stage ensures robust identification through the combined analysis of URL and HTML data. An extraction function ϕ(·) performs the feature extraction process for static analysis. Equation (5) defines the HTML feature collection process for static detection analysis.

(5) $$h_{0}\leftarrow \mathrm{GetHTML}\left(WV_{\mathrm{sandbox}}\right)$$

$h_{0}$ represents the initial state of the HTML, capturing information prior to any user actions such as button clicks or form submissions. Extraction of $h_{0}$ ensures the utilization of data available without additional execution, maintaining a fixed baseline for the static phase.

(6) $$x_{\mathrm{html}}\leftarrow \phi_{\mathrm{html}}\left(h_{0}\right)$$

Equation (6) subsequently describes the extraction of page structure-based features from $h_{0}$. The vector $x_{\mathrm{html}}$ incorporates various static attributes of the initial page, including input fields, forms, buttons, and link structures, to facilitate phishing identification.

(7) $$x_{\mathrm{url}}\leftarrow \phi_{\mathrm{url}}\left(u\right)$$

Equation (7) represents the extraction of static features from the URL string *u*. The feature vector $x_{\mathrm{url}}$ incorporates lexical information within *u*, including length, special character distribution, and domain patterns, to serve as an input for the static detection model. These URL-specific attributes facilitate the identification of suspicious string-level patterns during the preliminary assessment.

(8) $$x_{\mathrm{static}}\leftarrow \left[x_{\mathrm{html}}\;∥\;x_{\mathrm{url}}\right]$$

Equation (8) combines the extracted static features into a single vector $x_{\mathrm{static}}$ through the concatenation of $\left[x_{\mathrm{html}}\;∥\;x_{\mathrm{url}}\right]$. The concatenated vector serves as the input for the static detection model. Equation (9) presents the derivation of the phishing risk score $\theta_{\mathrm{static}}$ from the static classifier $f_{\mathrm{static}}$.

(9) $$\theta_{\mathrm{static}}\leftarrow f_{\mathrm{static}}\left(x_{\mathrm{static}}\right),\;\theta_{\mathrm{static}}\in \left[0,1\right]$$

The continuous static risk score $\theta_{\mathrm{static}}$ enables immediate classification for definitive cases while directing ambiguous inputs toward dynamic analysis. A dual-threshold structure, comprising $T_{\mathrm{allow}}$ and $T_{\mathrm{block}}$, is used as a routing mechanism in the staged detection pipeline rather than as a single arbitrary classification cut-off. The lower threshold $T_{\mathrm{allow}}$ is used to allow low-risk samples with high confidence, whereas the upper threshold $T_{\mathrm{block}}$ is used to block high-risk samples with high confidence. Samples located between the two thresholds are treated as ambiguous and are forwarded to the dynamic analysis stage. This design reduces the risk of directly allowing uncertain phishing samples, avoids unnecessary blocking of uncertain benign samples, and limits dynamic-analysis overhead by applying runtime observation only to ambiguous cases. The threshold setting is justified by the threshold-sweep analysis reported in the Section 4.3.

In WHALE, the two thresholds are interpreted according to their routing roles. Low-score samples correspond to high-confidence benign cases, high-score samples correspond to high-confidence phishing cases, and intermediate-score samples correspond to cases where static evidence is insufficient. These intermediate cases are not forced into an immediate static decision but are forwarded to dynamic analysis. Therefore, the dual-threshold design reflects the operational goal of WHALE: immediate decisions are made only for high-confidence cases, while uncertain cases are handled by runtime observation. Equation (10) defines the decision logic for the static stage.

(10) $$d_{\mathrm{static}}\left(\theta_{\mathrm{static}}\right)=\left\{\begin{array}{cc} \mathrm{BLOCK}, & \theta_{\mathrm{static}}\geq T_{\mathrm{block}}\\ \mathrm{ALLOW}, & \theta_{\mathrm{static}}\leq T_{\mathrm{allow}}\\ \mathrm{AMBIGUOUS}, & \mathrm{otherwise} \end{array}\right.$$

Equation (10) classifies the static assessment results into three distinct categories known as BLOCK, ALLOW, and AMBIGUOUS. $\theta_{\mathrm{static}}$ exceeding $T_{\mathrm{block}}$ triggers an immediate block identifying the input as phishing. Conversely, $\theta_{\mathrm{static}}$ below $T_{\mathrm{allow}}$ permits access and designates the URL as safe. The interval between these dual thresholds represents an area where static information remains insufficient for a definitive conclusion. Ambiguous outcomes are forwarded to subsequent dynamic analysis. Equation (11) defines the set of ambiguous inputs as $\Omega_{\mathrm{ambiguous}}$.

(11) $$\Omega_{\mathrm{ambiguous}}=\left\{\;u|T_{\mathrm{allow}}<\theta_{\mathrm{static}}<T_{\mathrm{block}}\;\right\}$$

Equation (11) defines $\Omega_{\mathrm{ambiguous}}$ as the set of URLs where static indicators remain inconclusive. When $\theta_{\mathrm{static}}$ falls within this range, WHALE initiates additional dynamic analysis. Otherwise, the system immediately blocks the input or forwards *u* to the *User WebView* based on the static result. This hierarchical decision structure is formally defined in Equation (12).

(12) $$d=\left\{\begin{array}{cc} d_{\mathrm{static}}\left(\theta_{\mathrm{static}}\right), & \theta_{\mathrm{static}}\notin \Omega_{\mathrm{ambiguous}}\\ d_{\mathrm{dynamic}}\left(\theta_{\mathrm{dynamic}}\right), & \theta_{\mathrm{static}}\in \Omega_{\mathrm{ambiguous}} \end{array}\right.$$

The selective activation of the dynamic detection stage for ambiguous instances within $\Omega_{\mathrm{ambiguous}}$ significantly decreases overall latency and computational overhead compared to full-scale dynamic analysis.

### 3.4. Dynamic Phishing Detection

The dynamic detection stage is executed only for inputs classified as uncertain by the static stage. This stage differs from a general webpage crawler because it does not aim to automatically discover credential-request pages across arbitrary webpages. WHALE instead targets credential-relevant pages or credential-request page (CRP) candidates reached inside the isolated *Sandbox WebView*. The purpose of this stage is to evaluate whether the credential submission flow is suspicious before real user credentials are released to the page. To achieve this, WHALE uses decoy credentials during dynamic analysis. ${\mathrm{State}}_{\mathrm{pre}}$ denotes the page state before decoy credential submission, and ${\mathrm{State}}_{\mathrm{post}}$ denotes the page state after decoy credential submission. WHALE enters decoy credentials, instead of real user credentials, into the detected credential fields inside the *Sandbox WebView*. It then triggers a controlled submit event and observes the post-submit page state. During this process, real user credentials are never inserted into the page DOM. WHALE determines the risk of the credential flow only from the state changes observed before and after decoy credential submission. The dynamic analysis does not use redirection or domain transition as standalone decision evidence. A phishing page may consume credentials while remaining on the same visible URL. At the same time, legitimate services may perform benign redirections during login, SSO, OAuth, or payment workflows. For this reason, WHALE does not rely on URL navigation itself. It jointly analyzes changes in form structure, credential roles, hidden inputs, response signals, handler mutations, and credential-consumption behavior before and after decoy credential submission.

The dynamic feature vector is defined in Equation (13). WHALE extracts state-transition features by comparing the pre-submit state ${\mathrm{State}}_{\mathrm{pre}}$ and the post-submit state ${\mathrm{State}}_{\mathrm{post}}$. The dynamic feature vector $x_{\mathrm{dynamic}}$ does not represent a single URL-change or domain-transition signal. Rather, it represents changes in form structure, credential roles, hidden inputs, response signals, handler mutations, and credential-consumption behavior.

(13) $$\begin{array}{cc} x_{\mathrm{dynamic}} & =\Phi ({\mathrm{State}}_{\mathrm{pre}},{\mathrm{State}}_{\mathrm{post}})\\ \; & =[x_{\mathrm{form}},x_{\mathrm{role}},x_{\mathrm{hidden}},x_{\mathrm{response}},x_{\mathrm{handler}},x_{\mathrm{consumption}}] \end{array}$$

In Equation (13), $x_{\mathrm{dynamic}}$ denotes the dynamic feature vector extracted from the state transition before and after decoy credential submission. The function Φ extracts state-transition features between the two page states. The vector $x_{\mathrm{dynamic}}$ consists of six feature families. The component $x_{\mathrm{form}}$ represents changes in the visible-form structure before and after submission. This feature family includes form fingerprint distance, form-count changes, visible-input-count changes, fillable-input-count changes, and visible-text changes. It captures whether the login form is preserved, removed, or transformed into another input structure after submission. The component $x_{\mathrm{role}}$ represents changes in the roles of credential input fields. This feature family includes changes in credential roles such as identifier, secret, OTP, and financial fields. For example, if identifier and secret fields exist before submission but the secret field disappears, or an OTP field appears after submission, this may indicate that the credential flow has moved to another step. Accordingly, $x_{\mathrm{role}}$ includes credential-role transition distance, credential-role consumption ratio, role preservation ratio, credential-role L1 delta, consumed role count, and added role count. The component $x_{\mathrm{hidden}}$ represents changes in hidden inputs. A phishing page may store decoy credentials in hidden fields or mutate hidden values after submission. The corresponding features include additions, removals, value mutations of hidden inputs, and whether a decoy value appears inside hidden fields. The component $x_{\mathrm{response}}$ represents response signals shown by the page after submission. A legitimate login page often presents validation signals such as invalid, required, wrong password, account not found, or retry requests when decoy credentials are incorrect.

A phishing page may instead consume the decoy credentials and move to another step or display a success-like screen without validation errors. Thus, $x_{\mathrm{response}}$ includes invalid warnings, required-field warnings, password errors, wrong-code messages, CAPTCHA and MFA indicators, same-credential retry requests, credential-form reappearance, success-like transitions, OTP-page transitions, and no-invalid-warning signals. The component $x_{\mathrm{handler}}$ represents changes in submission-handling structures such as form action, form method, submit handler, and onclick handler. JavaScript-based phishing pages or handler-mutation attacks may modify only the submission logic without changing the visible URL. The corresponding features include form-action changes, form-method changes, form-handler changes, submit-button onclick changes, visible-form fingerprint changes, and hidden-form fingerprint changes. The component $x_{\mathrm{consumption}}$ represents whether the decoy credentials are consumed after submission. A legitimate page tends to reject decoy credentials and preserve the same form or request re-entry. A phishing page may instead consume the decoy credentials, clear the input values, remove the credential form, or move to another credential step. Accordingly, $x_{\mathrm{consumption}}$ includes decoy-input clear ratio, secret clear ratio, identifier persistence ratio, post-submit retry consistency index, credential-flow consumption index, secret-only clear score, retry evidence score, and consume evidence score.

In this paper, the feature families in Equation (13) are instantiated as 59 state-transition features. These 59 features cover form, credential-role, hidden-input, response, handler, and consumption behavior before and after decoy credential submission. URL, domain, endpoint, HTTPS, localhost, IP address, and target-domain features are excluded from the dynamic feature vector. This exclusion prevents the model from learning environment-specific artifacts caused by differences between locally rehosted phishing pages and live benign websites. The dynamic phishing score is defined in Equation (14).

(14) $$\theta_{\mathrm{dynamic}}=f_{\mathrm{dynamic}}\left(x_{\mathrm{dynamic}}\right)$$

In Equation (14), $\theta_{\mathrm{dynamic}}$ denotes the phishing score computed by the dynamic analysis, and $f_{\mathrm{dynamic}}$ denotes the dynamic classifier. In the experiments of this paper, $f_{\mathrm{dynamic}}$ is a Histogram Gradient Boosting (HGB)-based classifier that receives the 59 state-transition features as input. The trained model is embedded in the Android application as a JSON tree ensemble. The Android-side evaluator computes $\theta_{\mathrm{dynamic}}$ using $x_{\mathrm{dynamic}}$ calculated inside the *Sandbox WebView*. The computation process of the HGB-based dynamic classifier is expressed in Equation (15).

(15) $$\theta_{\mathrm{dynamic}}=\mathrm{sigmoid}\left(\sum_{m=1}^{M}\psi_{m}\left(x_{\mathrm{dynamic}}\right)\right)$$

In Equation (15), $\psi_{m}$ denotes the output of the *m*-th boosted decision tree, and *M* denotes the number of boosted decision trees. In this paper, *M* is set to 120. The function sigmoid converts the accumulated tree-ensemble output into a phishing probability. Each boosted decision tree computes a partial risk score based on the state-transition features in $x_{\mathrm{dynamic}}$. WHALE aggregates all tree outputs to obtain the final dynamic phishing score $\theta_{\mathrm{dynamic}}$. The final dynamic decision is defined in Equation (16).

(16) $$d_{\mathrm{dynamic}}=\left\{\begin{array}{cc} \mathrm{BLOCK}, & \theta_{\mathrm{dynamic}}\geq \tau_{\mathrm{dynamic}}\\ \mathrm{ALLOW}, & \theta_{\mathrm{dynamic}}<\tau_{\mathrm{dynamic}} \end{array}\right.$$

In Equation (16), $\tau_{\mathrm{dynamic}}$ denotes the dynamic decision threshold. If $\theta_{\mathrm{dynamic}}$ is greater than or equal to $\tau_{\mathrm{dynamic}}$, WHALE classifies the page as phishing and blocks it. If $\theta_{\mathrm{dynamic}}$ is lower than $\tau_{\mathrm{dynamic}}$, WHALE regards the page as safe at the dynamic stage and may release the URL to the *User WebView* according to the staged decision policy.

Therefore, the dynamic stage evaluates credential-flow behavior from state-transition evidence rather than from URL or domain changes alone. Using decoy-only submission inside the *Sandbox WebView* and converting pre-submit and post-submit changes into 59 state-transition features, WHALE can analyze suspicious credential flows while avoiding exposure of real user credentials.

## 4. Results

In this section, simulations were conducted to evaluate the performance of the proposed WHALE framework. The setup introduces simulation environments and phishing scenarios designed according to a specific threat model. Static and dynamic detection performance was evaluated independently according to scenarios specific to each stage. The static stage evaluation measures static detection capabilities using features derived from URL strings and initial HTML structures. The dynamic detection-stage evaluation analyzes dynamic detection performance using observation logs collected after executing event scenarios.

### 4.1. Setup

The model training and conversion processes were conducted on a desktop environment equipped with an Intel Ultra 9 285K CPU, 128 GB DDR5 RAM, an NVIDIA RTX 5070 Ti GPU, and CUDA 12.8. The static model was implemented and trained using TensorFlow 2.21.0, and the trained Keras model was converted into a TensorFlow Lite model for mobile deployment [26]. The dynamic model was trained as an HGB-based classifier using 59 state-transition features and was exported as a JSON tree ensemble for execution inside the Android application. Android Studio was used for application development and preliminary functional verification. Android Studio Otter 2 Feature Drop (Version 2025.2.2 Patch 1) was adopted as the development environment. For the final mobile evaluation, runtime and resource overhead were measured on a real Android device, Samsung Galaxy S23 Ultra (SM-S918N), running Android SDK 36. The real-device measurements include static and dynamic runtime, model inference latency, CPU usage, memory changes, battery consumption, charge consumption, and model asset size. The experimental setup, therefore, separates desktop-based model training from real-device mobile evaluation.

### 4.2. Dataset

#### 4.2.1. Static Dataset

**Figure 3:**
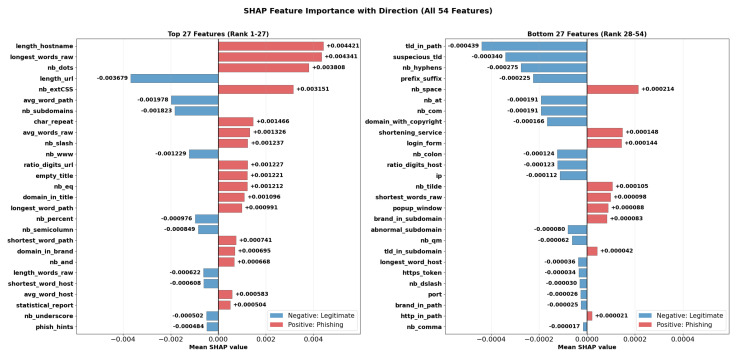
SHAP feature importance.

Static analysis was conducted using the “Web page phishing detection” dataset from Mendeley Data [38]. The dataset consists of 11,480 samples, including 5740 legitimate instances and 5740 phishing instances. Although this dataset is not a QR-image-specific dataset, it is consistent with the input scope of WHALE’s static stage. In a quishing scenario, the QR code acts as a delivery medium that embeds a URL, and the actual phishing judgment is performed after the embedded URL is decoded and the corresponding webpage is loaded. Therefore, the static stage of WHALE does not classify the visual QR image itself. Instead, it analyzes the decoded URL string and the initially loaded webpage content. For this reason, a webpage phishing dataset containing URL-level and page-level features is appropriate for evaluating the static analysis stage of WHALE. The Mendeley dataset contains 86 features in total, including URL lengths, hostname lengths, and IP address usage. Adapting to local mobile environments necessitated excluding 8 features requiring external API queries and 14 features unsupported within an Android WebView framework. Variables with constant values or negligible distribution showed zero importance, prompting SHapley Additive exPlanations (SHAP) analysis as depicted in Figure 3. Following the analysis, ten additional features were removed to enhance overall model efficiency [39].

The static features used in the simulation include URL lengths, hostname lengths, special character counts, and word length statistics. Such features exhibit significant distribution asymmetry and frequent outliers. Applying standard normalization techniques such as *StandardScaler* or *MinMaxScaler* to outlier features causes severe distortion.

Therefore, *RobustScaler* is employed during the training phase to mitigate the impact of outliers, utilizing the median and interquartile range to normalize the feature space. As a result, a total of 54 features were used in the simulation, as listed in Table 3.

#### 4.2.2. Dynamic Dataset

The dynamic dataset was constructed to evaluate credential-flow behavior observed inside the *Sandbox WebView*. The dynamic evaluation in this study does not target arbitrary webpages. Instead, it focuses on URLs classified as uncertain by the static analysis and reaching a CRP candidate. Each sample is executed inside the isolated *Sandbox WebView*, where WHALE enters decoy credentials instead of real user credentials and collects page-state changes before and after submission. The phishing samples were collected from DynaPD-based rehosted phishing kits [31,40], and the benign samples were collected from benign CRP pages. This evaluation includes only strict submission flows in which the decoy credential is observed in a credential-bearing POST payload. Therefore, the dataset targets CRP flows with observable decoy credential submission rather than arbitrary webpages. The dynamic dataset contains 2837 samples. Among them, 1472 samples are phishing, and 1365 samples are benign. The dataset was divided using a source-group-disjoint 60–40 held-out split. The training set contains 1697 samples, including 884 phishing samples and 813 benign samples. The test set contains 1140 samples, including 588 phishing samples and 552 benign samples. The train and test source-group overlap was confirmed to be zero.

To prevent the dynamic model from learning environment-specific artifacts, URL, domain, endpoint, HTTPS, localhost, IP address, and target-domain features were excluded from the dynamic feature vector. The occurrence of a POST request itself was also not used as a model feature and was used only as a condition for constructing the strict subset. The final dynamic model uses 59 state-transition features extracted from the page states before and after decoy credential submission. These features include form-structure changes, credential-role transitions, hidden-input mutations, response signals, handler mutations, and credential-consumption behavior.

### 4.3. Static Model Evaluation

Phishing detection simulations were conducted to evaluate the performance of the static detection model in WHALE, with comparative assessments against baseline models. Baseline models include machine learning-based classifiers presented in [41], comprising Logistic Regression (LR), Random Forest (RF), XGBoost (XGB), Explainable Boosting Machine (EBM), and Support Vector Machine (SVM). The performance evaluation of the models used standard metrics including *Accuracy*, *Precision*, *Recall*, the *F1-score*, and AUC.

To further strengthen the comparison between WHALE and recent state-of-the-art phishing and quishing detection methods, we expanded the reproduction-based comparison by adding two recent high-tier phishing detection baselines. The compared methods cover URL-only deep learning detection, mobile phishing ensemble detection, URL-based stacked generalization, QR image-only quishing detection, attribute-based URL detection, and transformer-based URL detection. The reproduced or adapted methods include Haq et al. 1D-CNN [42], Phish-Jam Super Learner [43], AntiPhishStack Meta-XGB [44], Trad and Chehab QR Pixel XGBoost [8], Aliyazicioglu-inspired URL-Attribute RF [45], and BERT-PhishFinder-style DistilBERT [46]. These methods were selected because they represent recent model families closely related to phishing or quishing detection while covering different input assumptions, including URL strings, mobile-oriented ensemble detection, stacked URL classification, QR image patterns, URL-derived attributes, and transformer-based URL representations. The comparison does not directly reuse the original reported performance values because the original studies used different datasets, input modalities, feature spaces, and evaluation protocols. Instead, the results in Table 4 report controlled reproduction or controlled-adaptation performance on the decoded-URL phishing dataset used for WHALE. Therefore, Table 4 should be interpreted as a controlled comparison under a unified dataset setting, rather than as a direct comparison with the original reported values of each paper.

**Figure 4:**
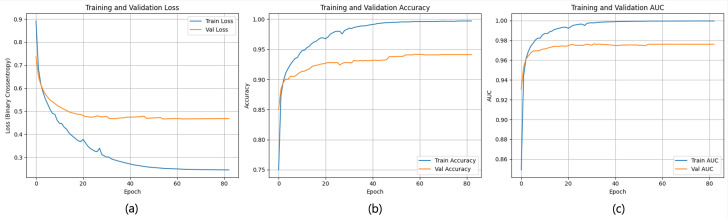
Model performance: (**a**) training and validation loss, (**b**) training and validation accuracy, and (**c**) training and validation AUC.

To ensure experimental reliability, the entire dataset was partitioned into 60% for training, 20% for validation, and 20% for testing. Average performance was derived from 10 random seed shuffles with two repetitions per seed, utilizing a batch size of 16 for up to 100 epochs. Early stopping was implemented to prevent overfitting, terminating training if validation loss failed to improve for 20 consecutive epochs. Figure 4 depicts the convergence characteristics observed during the training phase. The proposed model exhibits a consistent decrease in training loss with stable validation loss, leading to early stopping at an average of approximately 70 epochs.

Generalization was achieved by selecting model weights corresponding to the point of minimum validation loss. According to Figure 4b,c, accuracy and AUC demonstrate rapid initial growth up to 20 epochs, followed by steady convergence, confirming the training stability of the static detection model. Table 5 shows that WHALE outperforms the conventional machine learning baselines in terms of accuracy (93.86%), precision (93.08%), recall (94.78%), and F1-score (93.92%). In addition, Table 4 presents the reproduction-based and controlled-adaptation comparison with recent phishing and quishing detection methods. WHALE achieved an Accuracy of 0.9330, Precision of 0.9285, Recall of 0.9382, F1-score of 0.9333, and AUC of 0.9752. Among the reproduced methods, BERT-PhishFinder-style DistilBERT achieved the highest static classification performance, with an Accuracy of 0.9450, Precision of 0.9648, F1-score of 0.9438, and AUC of 0.9844. However, WHALE achieved higher Recall than BERT-PhishFinder-style DistilBERT, with 0.9382 compared with 0.9238. This is important in phishing detection because missed phishing pages directly increase user exposure risk. The Aliyazicioglu-inspired URL-Attribute RF baseline achieved an F1-score of 0.8968 and an AUC of 0.9604, showing that URL-derived attribute features provide useful phishing evidence but remain less effective than WHALE under the controlled dataset setting. The QR image-only method based on Trad and Chehab showed the largest performance gap, with an F1-score of 0.7794 and an AUC of 0.8621. These results show that transformer-based URL detection can provide very strong static classification performance, while WHALE remains competitive in static detection and provides higher phishing recall than the transformer baseline.

The comparison further clarifies the difference in detection scope between WHALE and existing approaches. As summarized in Table 1, scanner-based QR phishing methods mainly inspect URL-level static cues or blacklist and reputation information after QR scanning [30,32], while QR image-based methods classify maliciousness from QR matrix or pixel-level patterns before webpage execution [8,34,35]. Reference-based phishing detection improves phishing identification through visual or brand-reference verification, but it generally depends on reference availability and additional interaction or verification procedures [31]. In addition, the newly added URL-attribute and transformer-based baselines show that recent phishing URL detectors can achieve strong static classification performance on decoded URLs. In particular, the BERT-PhishFinder-style DistilBERT baseline outperformed the static WHALE classifier in Accuracy, Precision, F1-score, and AUC under the controlled static setting. However, WHALE achieved higher Recall than the BERT-PhishFinder-style DistilBERT baseline, with 0.9382 compared with 0.9238. This is important in phishing detection because missed phishing pages directly increase user exposure risk. The QR image-only method showed a larger performance gap, indicating that QR image patterns alone provide limited evidence for phishing judgment when compared with URL and page-based features extracted after QR payload decoding. Therefore, Table 4 should be interpreted as a static-stage comparison under controlled decoded-URL conditions. These static baselines do not execute the decoded URL inside a mobile Sandbox WebView, do not use decoy credential submission, and do not observe credential-flow state transitions after controlled submission. In contrast, WHALE is designed for the QR scanning workflow after URL decoding. The decoded URL is first executed inside an isolated Sandbox WebView, static analysis is applied to the URL and the initial page state, and dynamic analysis is invoked only for ambiguous cases. In the dynamic stage, WHALE uses decoy credentials instead of real user credentials and analyzes credential-flow state transitions before and after controlled submission. Therefore, the contribution of WHALE is not limited to static classification performance. It also lies in combining on-device QR-triggered execution isolation, selective dynamic triggering, and credential-flow-based behavioral analysis to reduce real user credential exposure.

**Figure 5:**
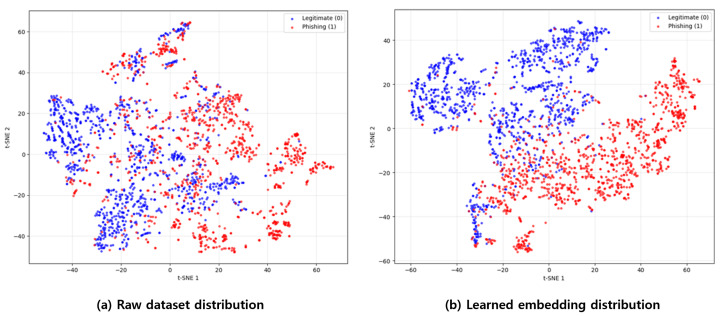
Embedding space visualization (t-SNE).

The static model is retained because it provides a continuous risk score for dual-threshold decision-making and selective dynamic triggering, rather than because of accuracy gain alone. Figure 5 illustrates the t-SNE visualization of the static feature space, demonstrating clear separability between phishing and benign samples. Raw dataset embeddings are illustrated in Figure 5a. Figure 5b shows that legitimate and phishing samples form distinct clusters, mixed only at identifiable boundary regions. Therefore, Figure 5 demonstrates that URL strings and initial HTML-based features provide sufficient discriminative ability for classification.

**Figure 6:**
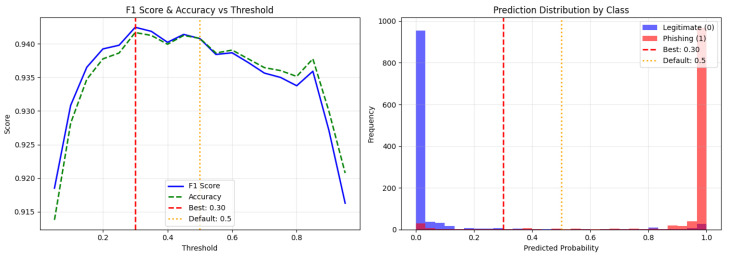
WHALE threshold and prediction distribution.

**Figure 7:**
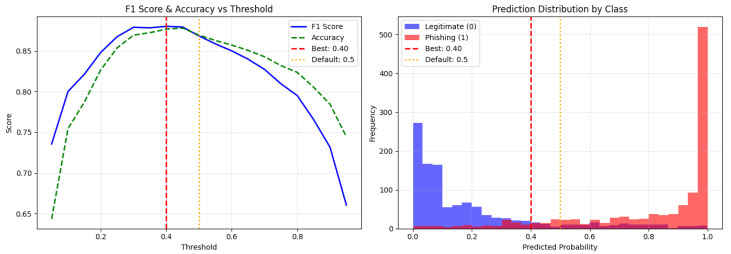
LR threshold and prediction distribution.

**Figure 8:**
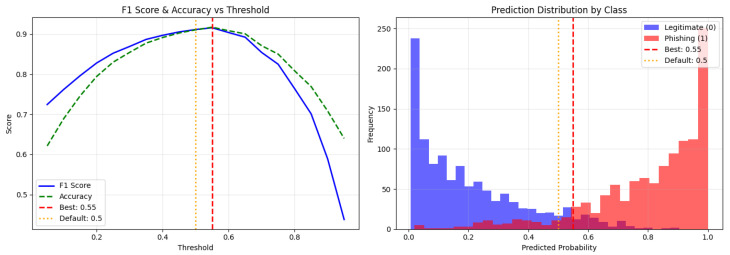
RF threshold and prediction distribution.

**Figure 9:**
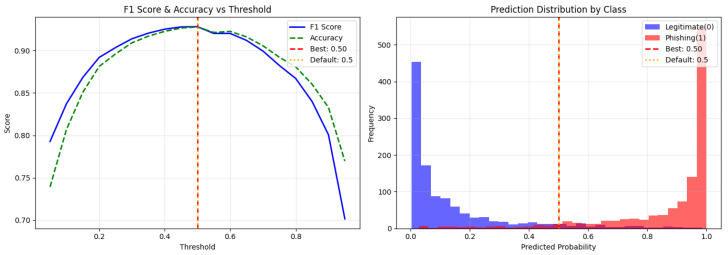
XGB threshold and prediction distribution.

**Figure 10:**
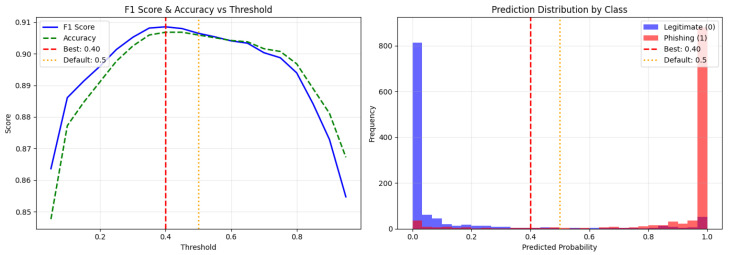
EBM threshold and prediction distribution.

**Figure 11:**
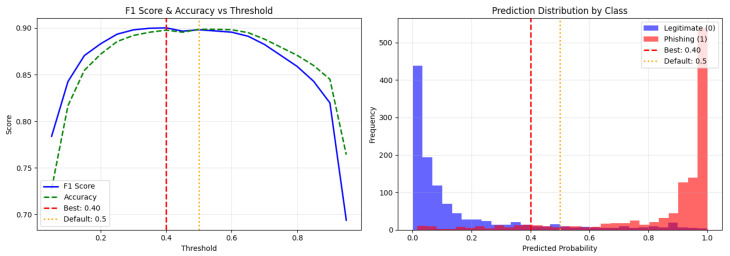
SVM threshold and prediction distribution.

However, boundary samples persist where feature values are ambiguous or identical, indicating inherent limitations in detecting phishing sites only through static features. Observed overlaps within the feature space necessitate more sophisticated detection mechanisms to effectively differentiate between legitimate and phishing classes. The experiments evaluated the impact of threshold variations on model performance and prediction probability distributions. Figure 6, Figure 7, Figure 8, Figure 9, Figure 10 and Figure 11 show threshold-dependent performance and distinct separation characteristics in prediction probabilities under uniform experimental conditions. These evaluations support the reliability of the static decision stage by showing how model performance changes across different threshold values, rather than relying only on a single fixed threshold. Figure 6 illustrates that both Accuracy and F1-score remain stable above 0.935 within the 0.3 to 0.75 threshold range, with 0.3 identified as the optimal single-threshold point.

This broad stable range supports the use of a dual-threshold design because the model output is not overly sensitive to a narrow threshold value. In the proposed staged framework, low-score samples can be treated as high-confidence benign cases, high-score samples can be treated as high-confidence phishing cases, and intermediate-score samples can be forwarded to dynamic analysis rather than being forced into an immediate static decision. Furthermore, the prediction probability distribution exhibits highly polarized separation at both extremes, demonstrating the model’s high confidence in distinguishing between classes. The distribution validates that the model successfully discriminates between classes using complex correlations identified among high-dimensional non-linear features. The relationship between thresholds and performance for baseline models was analyzed using the same setup, with results presented in Figure 6, Figure 7, Figure 8, Figure 9, Figure 10 and Figure 11. The distribution patterns of all comparative models were also analyzed. Figure 7 presents the threshold and prediction distribution for the LR model. The model yields an optimal threshold of 0.40 and maintains stable performance above 0.85. However, the mid-range shows significant mixing of legitimate and phishing samples. Such overlap causes the trade-off between false positives and negatives to shift easily, reflecting the limitations of simple linear combinations in capturing complex, non-linear features. Figure 8 presents the threshold and prediction distribution for the RF model. The model identifies an optimal threshold of 0.55 and exhibits the narrowest range where accuracy and F1-score exceed 0.9. However, the probability distribution shows a prolonged overlap between classes. The averaging process inherent in the ensemble structure causes this persistent overlap. Even when individual decision trees output extreme values, the combined predictions lead to a blended probability distribution across classes. In contrast, the XGB model achieves an optimal threshold of 0.50 and maintains stable performance above 0.9 over a broader range. However, certain regions still exhibit mixed class distributions due to underlying tree-based characteristics. Such iterative correction, inherent in gradient boosting, enhances class separation by incorporating weights from difficult-to-classify boundary samples. The threshold and prediction distribution for the XGB model are illustrated in Figure 9.

The threshold and prediction distribution for the EBM model are detailed in Figure 10. The model achieves an optimal threshold of 0.40 and maintains stable performance above 0.9 across a wide range from 0.25 to 0.7.

However, predicted probabilities separate strongly toward both extremes, similar to the WHALE results. Individual shape functions are learned and summed to assign high weights to features strongly associated with phishing. While this weighting allows for strong predictive contributions, the EBM architecture remains limited to pairwise interactions. Therefore, the model struggles to represent complex dependencies among the 54 features even when classification is successful.

Figure 11 presents the threshold and prediction distribution for the SVM model. The model identifies an optimal threshold of 0.40 and maintains relatively stable performance around 0.9. However, the class prediction distribution shows persistent overlap in the mid-range. The persistent mixing stems from the SVM objective of margin maximization rather than density estimation. While Platt scaling is applied, samples situated near the hyperplane do not reach the distributional extremes, resulting in a less polarized probability spread. Experimental results showed that predicted probability distributions polarizing toward endpoints achieved higher accuracy for class separation during the simulation. Moreover, threshold variation analysis based on *Accuracy* and *F1-score* confirmed the stability of performance for each model. WHALE exhibits a predicted probability distribution concentrated at both extremes, providing a wide and stable threshold range. This property is important for WHALE because the static score is used as a routing signal in the staged framework. A stable threshold range reduces sensitivity to a single fixed threshold and supports reliable separation between immediate decisions and ambiguous cases requiring dynamic analysis. Therefore, the embedding-based model is appropriate for the proposed static stage because it provides both competitive classification performance and stable risk scores for subsequent decision-making. Therefore, the results demonstrate that the static model of WHALE effectively captures complex interactions among multiple website features. The additional reproduction-based comparison further supports this finding. URL-only models can learn character-level or token-level patterns from URLs, but they cannot directly reflect page-level indicators such as HTML structure, login forms, form actions, and post-loading page behavior. QR image-only models can operate before URL extraction, but their available evidence is limited to QR matrix or pixel-level patterns.

In contrast, WHALE uses URL and page-based static features extracted after QR payload decoding, which provide stronger evidence for QR phishing detection under the same dataset conditions. However, the prediction probability distribution remains mixed in the ambiguous region, indicating that reliable judgment is difficult with static features alone. This ambiguity is mainly related to the inherent limitations of URL and initial HTML-based features. Static false positives can occur when benign webpages, such as login, payment, OAuth authentication, or account-management pages, contain long URLs, multiple query parameters, login forms, external resources, pop-up elements, or complex DOM structures that resemble phishing pages. Conversely, static false negatives can occur when phishing pages expose only weak evidence at the initial loading point using less suspicious URLs, HTTPS, legitimate-looking domain or path structures, minimal initial HTML, or interaction-gated credential fields. In such boundary cases, the initial URL and DOM snapshot may not provide sufficient evidence for a reliable phishing decision. These results support WHALE’s staged decision design, in which statically uncertain samples are forwarded to the dynamic-analysis stage rather than being immediately allowed or blocked.

### 4.4. Dynamic Detection Evaluation

The dynamic detection evaluation was conducted using a dynamic model with 59 credential-flow state-transition features. The evaluation used a source-group-disjoint split described in the Dynamic Dataset section, in which 60% of the dynamic dataset was used for training, and the remaining 40% was held out as an independent test set. The independent test set was not used for training and contained 1140 samples, including 588 phishing samples and 552 benign samples. The dynamic model used 59 state-transition features extracted from the page states before and after decoy credential submission.

The decision threshold of the dynamic detection stage was not selected from the independent test set. After training the model, we performed a threshold sweep on the phishing scores computed from the training split and selected the threshold that maximized the F1-score on the training split. The selected threshold was then fixed, and the source-group-disjoint independent test set was used only for final performance evaluation. The dynamic threshold used in this experiment was 0.4356. This procedure prevents test label information from being used during threshold selection and reduces bias in the final evaluation. The dynamic threshold selection process is expressed in Equation (17).

(17) $$\tau_{\mathrm{dynamic}}=\arg\max_{\tau \in Q_{\mathrm{train}}}F1_{\mathrm{train}}\left(\tau \right)$$

In Equation (17), $Q_{\mathrm{train}}$ denotes the set of threshold candidates evaluated on the training split. $F1_{\mathrm{train}}\left(\tau \right)$ denotes the F1-score computed on the training split when a specific threshold τ is applied. Therefore, $\tau_{\mathrm{dynamic}}$ is determined as the threshold that achieves the highest F1-score on the training split. The independent test set is not used to select $\tau_{\mathrm{dynamic}}$ and is used only for final performance evaluation with the fixed threshold.

Table 6 presents both the independent test results and the 10-run rolling group-disjoint auxiliary validation results of the dynamic detection stage. On the independent test set, WHALE achieved Precision of 0.895, Recall of 0.946, Specificity of 0.882, and F1-score of 0.920. The confusion matrix was TP = 556, FP = 65, TN = 487, and FN = 32, resulting in an Accuracy of 0.915. This means that WHALE correctly detected 556 out of 588 phishing samples as true positive cases, while 32 phishing samples were classified as false negative cases. Therefore, the false negative rate on the independent test set was approximately 5.4%. Recall of 0.946 shows that the dynamic detection stage detects phishing flows at a high rate. In particular, WHALE does not use redirection or domain transition as standalone decision evidence but jointly analyzes state transitions before and after decoy credential submission. Therefore, even if a page remains on the same visible URL after submission, WHALE can reflect it as a risk signal when credentials are consumed or when the form structure, credential roles, hidden inputs, response signals, handlers, or consumption behavior change. Precision of 0.895 indicates that most samples classified as phishing by WHALE were actual phishing samples. In the independent test set, 621 samples were classified as phishing, consisting of 556 TP cases and 65 false positive cases. This indicates that the dynamic detection stage actively detects phishing cases while limiting excessive blocking of benign pages to a controlled level. Specificity of 0.882 means that 487 out of 552 benign samples were correctly classified as TN cases.

This metric is important because normal pages can also produce redirections or state changes during login, SSO, OAuth, or payment workflows. Since WHALE considers credential-flow state changes before and after decoy credential submission rather than redirection itself, these signals help distinguish benign flows such as validation errors, retry behavior, and form preservation.

The F1-score of 0.920 shows the balance between Precision and Recall. In security detection, low Recall can expose users to phishing pages, whereas low Precision can increase unnecessary blocking of benign pages. WHALE achieved both Recall of 0.946 and Precision of 0.895 on the independent test set. This indicates that WHALE maintains a high phishing detection rate while controlling false positives from a usability perspective. The remaining errors further clarify the behavior of the dynamic state-transition model.

The 65 false positives indicate that some benign credential-request flows can still resemble phishing-like credential consumption after decoy credential submission. For example, normal login, OAuth, payment, identity-provider, or multi-step authentication workflows may update forms, modify the DOM, or move to another authentication step after submission. These behaviors can overlap with suspicious state-transition patterns even when the page is benign. The 32 false negatives indicate cases where phishing samples did not expose sufficiently distinctive state-transition signals during the bounded decoy-submission observation window. Such cases may occur when the submission logic is weakly observable, delayed, challenge-gated, or inactive in the rehosted environment. Therefore, the dynamic results should be interpreted as performance on strict decoy-observed CRP submission flows, while broader adversarial cases and limitations are discussed in the Security Analysis and Limitations sections.

The 10-run mean result in Table 6 serves as an auxiliary validation result showing that the performance does not depend on a single split. In the 10-run rolling 5:1:4 group-disjoint evaluation, the average test sample size was 1,134.8, and the model achieved an average Precision of 0.895, Recall of 0.915, Specificity of 0.882, and F1-score of 0.904. In addition, ROC-AUC was 0.966, and PR-AUC was 0.968. This indicates that phishing flows and benign flows are stably separated not only in the final threshold-based decision but also from a score-ranking perspective. The results in Table 6 show that the dynamic detection stage of WHALE can effectively detect phishing behavior based on credential-flow state transitions before and after decoy credential submission. WHALE achieved high Recall and F1-score on the independent test set and maintained stable performance in the 10-run rolling group-disjoint evaluation. Therefore, the dynamic detection stage of WHALE can determine phishing risk based on actual credential-flow changes.

### 4.5. On-Device Runtime and Resource Overhead

To evaluate the practical feasibility of WHALE in an On-Device environment, we measured the runtime and mobile resource overhead of both the static and dynamic-analysis stages on a real Android device. The evaluation device was a Samsung Galaxy S23 Ultra (SM-S918N) equipped with the Qualcomm Snapdragon 8 Gen 2 Mobile Platform for Galaxy and 12 GB LPDDR5X RAM, running Android SDK 36. The measurements were conducted on the same independent test set used in the dynamic detection evaluation, consisting of 1140 samples with 588 phishing samples and 552 benign samples. In the runtime evaluation, we separated page-loading time from the internal analysis time of WHALE. Page-loading time can be affected by network conditions, server response latency, and webpage complexity. Therefore, the computational cost of WHALE should be interpreted mainly through feature extraction, model inference, and decision time. However, in the dynamic-analysis stage, some intervals inevitably include webpage-response factors such as WebView interaction, DOM updates, server responses, and redirection, because WHALE must observe the page behavior after decoy credential submission.

Table 7 shows the On-Device runtime overhead of the static and dynamic-analysis stages. In the static stage, the internal subtotal was 58.75 ms on average and 53.70 ms at the median. Most of the static overhead came from feature extraction, which took 55.25 ms on average and 50.30 ms at the median.

This is because WHALE computes 54 static features, including URL-string features, DOM traversal features, and HTML element inspection features. For some pages, the maximum feature extraction time reached 616.68 ms due to complex DOM structures or a large number of HTML elements. Thus, this overhead is better interpreted as page-structure-dependent feature computation cost rather than model inference cost.

The static model input and inference time were 3.49 ms on average and 3.35 ms at the median. This accounts for only a small portion of the total static analysis overhead. This result is expected because the static model of WHALE is implemented as a lightweight TFLite model for mobile deployment. Once the feature vector is constructed, the model only performs inference. In addition, WHALE does not rely on external server queries or blacklist lookups in the static stage, so network round-trip latency is not included in the model inference time. The final threshold decision took 0.01 ms on average because it only routes the model output score into ALLOW, BLOCK, or AMBIGUOUS through a simple comparison. The dynamic analysis stage is executed only when the static stage cannot make a confident decision. In this stage, WHALE does not use real user credentials. Instead, it performs a controlled submission flow with decoy credentials inside the Sandbox WebView. In Table 7, the CRP detection point indicates the time at which a credential page or form is observed. This value is not directly included in the dynamic internal subtotal. Rather, it represents the time required for the WebView to reach a usable CRP candidate state. The CRP detection point took 1473.56 ms on average and 1145.42 ms at the median. For some pages, it increased up to 21,948.73 ms due to delayed rendering or script-driven DOM updates. Therefore, this value should be interpreted as the WebView observation time required for the page to become credential-input-ready, not as the computational cost of the WHALE model. After a CRP was observed, the decoy probe after the CRP stage took 1572.52 ms on average and 1525.27 ms at the median. In this stage, WHALE identifies credential fields, inserts decoy credentials instead of real user credentials, and captures the pre-submit state. Thus, this interval is not a simple model-computation interval. It is the process of constructing the reference state required to compare the pre-submit and post-submit transitions. The submit after probe stage triggers the submit event after decoy credentials are inserted. This stage took 535.15 ms on average and 517.57 ms at the median. The submit event simulates a real user submission action, such as a button click or an enter-key event. It serves as the key transition point connecting the pre-submit state and the post-submit state in the dynamic analysis. After submission, WHALE performs the State59 observation stage. State59 observation took 2049.06 ms on average and 2315.42 ms at the median, which was the largest component of the dynamic-analysis stage. This stage compares the page states before and after decoy credential submission and converts the observed changes into 59 state-transition features. These features include form-structure changes, credential-role changes, hidden-input changes, response signals, handler mutations, and credential-consumption behavior.

The relatively large overhead of State59 observation is caused by the need to observe webpage responses after submission, including server responses, redirections, DOM updates, and delayed rendering. The HGB inference-only time was 7.74 ms on average and 1.12 ms at the median. This is very small compared with the dynamic internal subtotal of 4165.42 ms on average. Therefore, the latency of the dynamic analysis is not caused by heavy model inference. Instead, it mainly comes from safely reproducing the decoy credential submission flow inside the Sandbox WebView and observing the resulting page-state changes. Because the HGB model is a lightweight tree ensemble that receives the State59 features as input, it can perform inference within a short time on the Android device.

The final decision after State59 took 8.69 ms on average and 2.74 ms at the median. In this interval, WHALE compares the phishing score computed by the HGB model with the decision threshold and determines BLOCK or ALLOW.

The HGB inference-only time is separately reported as a model-only value inside the final decision interval. Therefore, HGB inference-only and final decision after State59 should not be added as independent intervals. The HGB inference-only value is reported to isolate the pure model inference cost, while the dynamic internal subtotal already includes the final decision after State59. Therefore, the dynamic internal subtotal was 4165.42 ms on average and 4691.51 ms at the median. The dynamic analysis requires a longer runtime than the static analysis. However, this overhead is not caused by large-scale model computation. It is the cost of validating the credential submission flow using decoy credentials inside the Sandbox WebView before releasing real user credentials. In particular, most of the dynamic overhead comes from decoy probing and State59 observation, while the dynamic HGB inference itself accounts for only a small portion of the total overhead.

In terms of model asset size, WHALE also has a structure suitable for mobile deployment. As shown in Table 8, the total model asset size of the PhishHunter-XLD ensemble is 256.718 MB. In contrast, the model asset size of WHALE, including the static TFLite model and the dynamic HGB JSON model, is 0.681 MB. Therefore, the PhishHunter-XLD ensemble requires approximately 377 times larger storage footprint than WHALE. This shows that WHALE reduces both inference latency and model footprint for On-Device QR phishing detection. We additionally compared the model inference cost of WHALE with that of the PhishHunter-XLD ensemble [47]. The comparison was conducted on the same Android device and was limited to model inference-only time.

This scope was used because page loading, WebView interaction, decoy credential input, submit events, and State59 observation can be affected by network conditions and webpage responses. Therefore, Table 9 compares only the computational cost required for model inference in the On-Device environment, excluding such external factors. The PhishHunter-XLD ensemble consists of XGBoost, char-LSTM, and DistilBERT. As shown in Table 9, the total ensemble inference time of PhishHunter-XLD was 118.341 ms on average and 115.953 ms at the median. Specifically, XGBoost inference took 0.422 ms on average, and char-LSTM inference took 1.566 ms on average. However, DistilBERT inference took 116.353 ms on average, accounting for most of the total ensemble inference time. This shows that the inference latency of the PhishHunter-XLD ensemble is dominated by the transformer-based DistilBERT model. In contrast, the model inference time of WHALE was much smaller. Static TFLite inference took 0.024 ms on average, and dynamic HGB inference took 0.063 ms on average.

Even when both static and dynamic model inference times were combined, the average inference time was only 0.088 ms. Thus, under the model inference-only comparison, the PhishHunter-XLD ensemble required more than 1.3 thousand times longer inference time than the static and dynamic model inference of WHALE. This result is achieved because WHALE uses a lightweight TFLite model for static analysis and an HGB tree ensemble that takes State59 features as input for dynamic analysis.

Mobile resource overhead was measured in terms of CPU usage, memory changes, and battery consumption. Table 10 shows the resource overhead of the static and dynamic-analysis stages, and Table 11 shows the integrated static and dynamic resource overhead. The static analysis stage recorded an average CPU time of 720.133 ms, with a median of 531.500 ms, whereas the dynamic-analysis stage recorded an average CPU time of 1409.766 ms, with a median of 1142.500 ms. When the static and dynamic stages were executed together, the average accumulated CPU time was 2129.899 ms. In this measurement, CPU time refers to the accumulated CPU processing time used by the Android process, not the elapsed wall-clock runtime. For CPU utilization, the static phase showed 54.493% one-core CPU utilization and 6.812% all-core CPU utilization on average. In contrast, the dynamic phase showed 19.220% one-core CPU utilization and 2.403% all-core CPU utilization on average. The static analysis concentrates on feature extraction and model inference within a short interval, resulting in higher one-core CPU utilization. The dynamic analysis has a longer runtime, but it includes waiting intervals for WebView interaction, page transition, and post-submit observation.

For this reason, its average CPU utilization is lower than that of the static phase. The integrated one-core CPU utilization was 20.411% on average, and the integrated all-core CPU utilization was 2.551% on average. This indicates that the dynamic analysis of WHALE does not continuously occupy the CPU as a heavy-computation pipeline, even though it includes a longer observation interval. Memory overhead was measured using Android Proportional Set Size (PSS) change, Java heap change, and native heap allocated change. In the static phase, the PSS change was −0.139 MB on average, and the Java heap change was −0.164 MB on average. In the dynamic phase, the PSS change was 0.142 MB on average, and the Java heap change was 0.174 MB on average.

When static and dynamic stages were integrated, the PSS change was only 0.003 MB on average, and the Java heap change was only 0.010 MB on average. In Android environments, memory change can be affected by garbage collection, WebView internal memory management, and OS background memory reclaim. Therefore, the near-zero integrated average is more important than the sign of each phase-level change. These results show that WHALE does not cause continuous memory growth during the analysis process. The native heap allocated change was 29.655 KB on average in the static phase and 162.277 KB on average in the dynamic phase. The integrated native heap change was 191.932 KB on average. The dynamic phase shows a larger native heap change because event execution, DOM snapshots, and State59 observation inside the Sandbox WebView require additional native allocations related to WebView and JavaScript bridge operations. However, the integrated native heap increase is much smaller than 1 MB, so it is not large enough to be considered a burdensome memory overhead on mobile devices. Battery consumption was measured using battery percentage change and charge change. In the static phase, the average battery percentage change was −0.011%, and the average charge change was −0.404 mAh. In the dynamic phase, the average battery percentage change was −0.017%, and the average charge change was −0.875 mAh. When static and dynamic stages were integrated, the average per-sample battery percentage change was −0.027%, and the average charge change was −1.278 mAh. Because Android battery counters are coarse-grained, median values often appear as 0 at the per-sample level. Therefore, battery overhead should be interpreted based on cumulative benchmark-level consumption rather than instantaneous per-sample changes.

The resource measurements show that WHALE has a practical overhead level for real mobile deployment, even when dynamic analysis is included. The dynamic stage requires longer runtime and CPU time than the static stage, but this is mainly due to the security-oriented observation of decoy credential submission inside the Sandbox WebView, not due to heavy model computation. In the model inference-only comparison, the static and dynamic inference time of WHALE was only 0.088 ms, and the model asset size was only 0.681 MB. The integrated resource measurements also showed very small memory changes, with 0.003 MB PSS change, 0.010 MB Java heap change, and 191.932 KB native heap change on average. Therefore, WHALE does not continuously occupy the CPU or cause large memory growth, even with dynamic analysis. These results indicate that the mobile resource requirements of WHALE are practical for On-Device QR phishing detection. From a scalability perspective, WHALE is designed to avoid executing dynamic analysis for every scanned QR URL. The static stage first separates high-confidence allow and high-confidence block cases using the dual-threshold policy, and the dynamic stage is invoked only when the static score falls into the ambiguous region. Therefore, the runtime of one QR scan depends on the routing result. When a QR URL is confidently classified by the static stage, WHALE requires only the static internal analysis time, which was 58.75 ms on average, corresponding to approximately 0.06 s per scan. In contrast, when a QR URL is routed to dynamic analysis and reaches a credential-request page candidate, WHALE performs additional security verification inside the Sandbox WebView. The average dynamic internal runtime was 4165.42 ms, corresponding to approximately 4.17 s per ambiguous credential-related scan. The 4.17 s dynamic verification time is mainly spent on WebView-based security observation rather than model computation. Specifically, after a credential-request page candidate is observed, decoy probing required 1572.52 ms, corresponding to approximately 1.57 s, controlled submission required 535.15 ms, corresponding to approximately 0.54 s, and State59 observation required 2049.06 ms, corresponding to approximately 2.05 s. In contrast, the HGB inference itself required only 7.74 ms, corresponding to approximately 0.008 s. This indicates that the scalability bottleneck is not the classifier, but the need to safely reproduce and observe the credential-flow transition before releasing real user credentials. The CRP detection point was reported separately because it reflects the time required for the WebView to reach a credential-input-ready state, which can be affected by page rendering, JavaScript execution, server response, and DOM updates. The average CRP detection point was 1473.56 ms, corresponding to approximately 1.47 s. If this CRP reaching time is also included, an ambiguous credential-related QR scan requires approximately 5638.98 ms, corresponding to approximately 5.64 s on average. Therefore, the practical interpretation of WHALE is that high-confidence static cases can be processed in approximately 0.06 s, whereas ambiguous credential-related cases require approximately 4.17 s of additional dynamic verification after CRP detection, or approximately 5.64 s when the CRP reaching time is included. Under continuous mobile usage or frequent QR scanning, repeated dynamic analysis may increase user-perceived latency, especially when many ambiguous QR codes are scanned consecutively or when pages intentionally delay rendering, submission responses, or DOM updates. However, this overhead is incurred only for uncertain cases that require runtime verification before real credential exposure. In practice, the overhead can be mitigated by applying dynamic analysis selectively, caching recent decisions for repeated URLs, limiting repeated analysis for the same host within a short time window, and presenting the dynamic stage as a pre-access security verification step for ambiguous QR scans. Therefore, the current implementation is most suitable for user-facing QR security verification in which static-only cases are processed quickly, and ambiguous credential-related pages are analyzed for several seconds before the user is allowed to enter real credentials. Further optimization of observation windows, caching policies, and asynchronous user-interface handling will be considered in future work to improve usability under continuous mobile usage.

## 5. Security Analysis

This paper considers a phishing scenario as the threat model. In this scenario, a webpage loaded in a WebView after QR code scanning attempts to steal user credentials. The proposed method performs staged analysis to detect and block phishing threats before the malicious page affects the user session. After *u* is obtained from the QR code, WHALE loads the input *u* into a *Sandbox WebView* isolated from the *User WebView*. The independent configuration of the *Sandbox WebView* prevents the sharing of cookies, storage, cache, autofill information, and login sessions with the *User WebView*. This isolated architecture prevents compromise of the user environment by restricting all session data to the sandbox context. Information accessible to an attacker remains confined to the sandbox context even when a malicious page is loaded.

The isolated structure also reduces secondary threats such as session hijacking and state tracking. In the static stage, static features are extracted from *u* based on the URL string and the initial HTML. Using the extracted features, the embedding-based deep learning model learns interactions among features. The trained model computes the phishing probability for an unidentified *u* to determine the maliciousness of the target. Static analysis depends on a snapshot at the observation point. This dependence makes static analysis vulnerable to data bias. Moreover, conditionally exposed behaviors, including cloaking, malicious actions triggered by user events, and multi-step redirections, remain difficult to capture through static analysis. Thus, the limitations of static detection are complemented through a dynamic detection stage that considers user interactions and post-event behavior. In the behavior-based dynamic analysis process, WHALE executes sandboxed decoy probing instead of releasing real user credentials. After decoy credential submission, WHALE compares the pre-submit and post-submit states to determine whether the credential-flow behavior is suspicious. The dynamic analysis does not treat redirection or domain transition as standalone evidence.

Instead, it analyzes credential-role transitions, form and handler mutations, hidden-input changes, validation or retry signals, and credential-consumption behavior. To clarify the security coverage of WHALE, we analyze representative adversarial cases. Table 12 summarizes each adversarial case, the attacker strategy, the runtime signals observed by WHALE, and the remaining limitations. For delayed redirection, WHALE observes post-submit state changes and delayed credential-flow transitions rather than relying only on immediate URL changes. For same-page credential harvesting, WHALE analyzes whether the page consumes decoy credentials, removes the credential form, mutates hidden inputs, or moves to another credential step without visible redirection. For JavaScript obfuscation and handler mutation, WHALE observes changes in form handlers, submit handlers, hidden fields, and post-submit DOM state transitions. For cloaking and interaction-gated phishing, WHALE performs controlled interaction in the *Sandbox WebView* before releasing real credentials. For CAPTCHA and MFA-gated pages and Sandbox WebView-aware evasion, WHALE treats the case as a limitation when the decoy credential submission flow cannot be observed reliably.

The adversarial cases in Table 12 can affect WHALE in different ways. Cloaking, user-agent filtering, and Sandbox WebView-aware evasion may increase false negatives because malicious behavior can be suppressed during sandbox execution. Very long delayed redirection or delayed DOM mutation can also reduce detection coverage when the malicious transition occurs outside the observation window. CAPTCHA- or MFA-gated phishing can limit the applicability of the dynamic classifier when decoy credential submission cannot be reliably observed. In contrast, benign SSO, OAuth, payment, or multi-step authentication workflows may produce state transitions that resemble credential consumption, which can increase false positives. Therefore, WHALE reduces user exposure by combining sandbox isolation, decoy-only probing, and credential-flow state-transition analysis, but it does not eliminate adaptive evasion risks. These remaining risks are further discussed in the Limitations and Generalizability section.

In the proposed staged phishing detection framework, when the analyzed *u* is classified as safe, it is delivered to the *User WebView*. When *u* is classified as phishing, WHALE blocks access and notifies the user of the potential threat through a warning interface. By isolating suspicious pages, using decoy-only probing, and blocking risky credential-flow behavior, WHALE reduces user exposure to both static and behavior-based dynamic quishing threats.

## 6. Limitations and Generalizability

WHALE shows practical detection performance in the evaluated setting, but several limitations remain. The generalizability of the proposed framework to unseen real-world QR phishing campaigns should be interpreted with caution. The dynamic evaluation in this study focuses on credential-request page flows in which decoy credential submission can be reliably observed inside the Sandbox WebView. Therefore, the reported dynamic performance should not be interpreted as complete coverage of all QR-based threats. Non-credential QR scams, pages that do not expose credential forms during the observation window, backend-dead phishing kits, CAPTCHA- or MFA-gated flows, and highly adaptive campaigns that suppress malicious behavior under sandbox execution remain outside the main binary dynamic evaluation.

The dynamic features of WHALE were designed to reduce dependence on environment-specific artifacts, but they cannot eliminate all dataset and deployment bias. In the dynamic model, URL, domain, endpoint, HTTPS, localhost, IP address, and target-domain features were excluded from the feature vector. Instead, WHALE analyzes credential-flow state transitions observed before and after decoy credential submission. This design can improve robustness against simple changes in hosting infrastructure, domain names, and redirection patterns. However, real-world phishing campaigns may vary across brands, languages, regions, hosting providers, JavaScript frameworks, authentication workflows, and temporal attack strategies. As a result, phishing pages whose observable behavior differs substantially from the evaluated CRP flows may lead to false negatives, whereas benign multi-step authentication flows that resemble credential consumption may lead to false positives. The dynamic-analysis stage introduces additional runtime overhead compared with static-only inspection. WHALE executes dynamic analysis only for ambiguous cases, and the model inference cost itself is lightweight. However, the dynamic stage still requires WebView interaction, decoy credential insertion, controlled submission, and post-submit state observation. Therefore, under continuous mobile usage or frequent QR scanning, repeated dynamic analysis may increase user-perceived latency. This overhead is mainly caused by security-oriented observation inside the Sandbox WebView rather than heavy model computation. Nevertheless, long-delay pages, complex DOM structures, slow server responses, and script-driven rendering can increase the observation time. WHALE also does not guarantee complete prevention against adaptive adversarial evasion. Attackers may attempt to detect sandbox execution, delay malicious behavior beyond the observation window, hide credential submission behind CAPTCHA or MFA challenges, or imitate benign validation and retry behavior. In such cases, malicious behavior may not be sufficiently exposed during sandbox execution, which can increase the risk of false negatives. Conversely, legitimate services using SSO, OAuth, payment authentication, OTP transitions, or multi-step login procedures may produce state transitions similar to credential consumption, which can increase the risk of false positives.

Therefore, WHALE should be interpreted as an On-Device QR phishing detection framework that reduces user exposure to credential-harvesting flows, rather than as a complete solution for all QR-related attacks. Future work will extend the evaluation to larger and continuously updated real-world QR phishing campaigns collected across different time periods, regions, service categories, and mobile environments. Additional evaluation will also include more diverse benign authentication workflows, non-credential QR scams, CAPTCHA- and MFA-gated cases, and adaptive sandbox-aware phishing pages to further validate the practical generalizability of the framework.

## 7. Conclusions

This paper proposed WHALE, an On-Device multi-stage phishing detection framework based on an isolated *Sandbox WebView* for QR-code-triggered phishing threats. WHALE does not directly deliver a QR-decoded URL to the *User WebView*. Instead, the URL is first analyzed inside the *Sandbox WebView*, which separates the detection process from the user session. This design prevents the scanned page from directly accessing user-side cookies, session information, storage, cache, autofill data, and login state during analysis. WHALE combines static analysis and dynamic analysis in a staged manner. In the static stage, WHALE computes a phishing risk score using 54 features extracted from URL strings and initial HTML structures. A dual-threshold decision policy is then used to classify high-confidence benign and phishing cases quickly. Inputs that remain uncertain after static analysis are forwarded to the dynamic stage. In the dynamic stage, WHALE does not use real user credentials. Instead, it inserts decoy credentials inside the *Sandbox WebView*, triggers a controlled submission event, and observes the page states before and after submission. The dynamic model then evaluates the credential-flow behavior using 59 state-transition features, including form-structure changes, credential-role transitions, hidden-input mutations, response signals, handler mutations, and credential-consumption behavior.

The experimental results show that the static model achieved an Accuracy of 93.86%, Precision of 93.08%, Recall of 94.78%, and F1-score of 93.92%. The dynamic detection stage achieved an Accuracy of 0.915, Precision of 0.895, Recall of 0.946, Specificity of 0.882, and F1-score of 0.920 on the source-group-disjoint independent test set. The 10-run rolling group-disjoint evaluation further showed stable performance, with an average F1-score of 0.904, ROC-AUC of 0.966, and PR-AUC of 0.968. These results indicate that WHALE can detect QR-based phishing threats by combining static risk scoring with credential-flow-based dynamic behavior analysis. The real-device evaluation also confirmed that WHALE can operate with practical overhead in a mobile environment. The internal subtotal of the static analysis stage was 58.75 ms on average, while the internal subtotal of the dynamic-analysis stage was 4165.42 ms on average. The dynamic overhead mainly comes from security-oriented observation inside the *Sandbox WebView*, such as decoy credential probing, submission, and State59 observation, rather than heavy model inference. In the inference-only comparison, the combined static and dynamic model inference time of WHALE was only 0.088 ms on average, and the model asset size was 0.681 MB. The integrated resource measurements also showed small memory changes and practical CPU and battery overhead on the real Android device. Therefore, WHALE protects the user session by isolating the QR-triggered execution path and improves phishing detection by combining static URL and page evidence with dynamic credential-flow evidence. The dynamic stage does not rely solely on redirection or domain transition. Instead, it determines phishing risk from actual state transitions before and after decoy credential submission. This enables WHALE to analyze suspicious credential flows while avoiding exposure of real user credentials.

Future work will extend the generality of the dynamic detection stage by incorporating more diverse real-world QR phishing cases and complex credential-flow scenarios. Additional research will also address CAPTCHA and MFA-gated flows, advanced cloaking, and Sandbox WebView-aware evasion. Furthermore, long-term evaluations across different mobile devices, Android versions, and network environments will be conducted to further validate the practical deployability of WHALE.

## Figures and Tables

**Table 2:** Notation.

Symbol	Meaning	Symbol	Meaning
qr	QR image	*u*	decoded URL
QRDecode(·)	QR decoder	A	attacker
g(·)	phishing detection function	$\hat{y}\left(u\right)$	phishing prediction for *u*
$U_{\mathrm{phishing}}$	phishing URL set	$U_{\mathrm{legitimate}}$	legitimate URL set
Pr[·]	probability	⌀	empty set
$WV_{\mathrm{user}}$	User WebView	$WV_{\mathrm{sandbox}}$	sandbox WebView
State(·)	WebView state	∩	intersection
InitSandboxWebView(·)	sandbox initialization function	Load(·)	URL loading function
GetHTML(·)	HTML extraction function	$h_{0}$	initial HTML state
$\phi_{\mathrm{url}}\left(\cdot \right)$	URL feature extractor	$\phi_{\mathrm{html}}\left(\cdot \right)$	HTML feature extractor
$x_{\mathrm{url}}$	URL feature vector	$x_{\mathrm{html}}$	HTML feature vector
$x_{\mathrm{static}}$	static feature vector	$f_{\mathrm{static}}\left(\cdot \right)$	static classifier
$\theta_{\mathrm{static}}$	static phishing risk score	$d_{\mathrm{static}}\left(\theta_{\mathrm{static}}\right)$	static decision
$T_{\mathrm{allow}}$	allow threshold	$T_{\mathrm{block}}$	block threshold
$\Omega_{\mathrm{ambiguous}}$	ambiguous region	*d*	final decision
${\mathrm{State}}_{\mathrm{pre}}$	page state before decoy credential submission	${\mathrm{State}}_{\mathrm{post}}$	page state after decoy credential submission
Φ(·,·)	state-transition feature extraction function	$x_{\mathrm{dynamic}}$	dynamic feature vector
$x_{\mathrm{form}}$	form-structure transition features	$x_{\mathrm{role}}$	credential-role transition features
$x_{\mathrm{hidden}}$	hidden-input transition features	$x_{\mathrm{response}}$	response-signal features
$x_{\mathrm{handler}}$	handler-mutation features	$x_{\mathrm{consumption}}$	credential-consumption features
$f_{\mathrm{dynamic}}\left(\cdot \right)$	dynamic classifier	$\theta_{\mathrm{dynamic}}$	dynamic phishing score
$\psi_{m}\left(\cdot \right)$	output of the *m*-th boosted decision tree	*M*	number of boosted decision trees
$\tau_{\mathrm{dynamic}}$	dynamic decision threshold	$d_{\mathrm{dynamic}}\left(\theta_{\mathrm{dynamic}}\right)$	dynamic decision
$Q_{\mathrm{train}}$	threshold candidate set on the training split	$F1_{\mathrm{train}}\left(\tau \right)$	training F1-score at threshold τ
ALLOW	allow decision	BLOCK	block decision
AMBIGUOUS	ambiguous decision	CRP	credential-request page

**Table 3:** Feature descriptions.

Feature	Description	Feature	Description
length_url	URL length	nb_subdomains	Number of subdomains
length_hostname	Hostname length	prefix_suffix	Hyphenated domain pattern
ip	IP address included in URL (0/1)	shortening_service	URL shortening service indicator (0/1)
nb_dots	Number of dots	length_words_raw	Number of URL tokens
nb_hyphens	Number of hyphens	char_repeat	Character repetition score
nb_at	Number of ‘@’ symbols	shortest_words_raw	Minimum token length in the URL
nb_qm	Number of ‘?’ symbols	shortest_word_host	Minimum token length in the hostname
nb_and	Number of ‘&’ symbols	shortest_word_path	Minimum token length in the path
nb_eq	Number of ‘=’ symbols	longest_words_raw	Maximum token length in the URL
nb_underscore	Number of ‘_’ symbols	longest_word_host	Maximum token length in the hostname
nb_tilde	Use of ‘~’ symbol (0/1)	longest_word_path	Maximum token length in the path
nb_percent	Number of ‘%’ symbols	avg_words_raw	Average token length in the URL
nb_slash	Number of slashes	avg_word_host	Average token length in the hostname
nb_colon	Number of colons	avg_word_path	Average token length in the path
nb_comma	Number of commas	phish_hints	Number of phishing-related keyword hits
nb_semicolumn	Number of semicolons	domain_in_brand	Whether the domain matches a known brand (0/1)
nb_space	Number of spaces or %20 tokens	brand_in_subdomain	Whether a brand name appears in the subdomain (0/1)
nb_www	Number of ‘www’ tokens	brand_in_path	Whether a brand name appears in the path (0/1)
nb_com	Number of ‘com’ tokens	suspicious_tld	Suspicious TLD indicator (0/1)
nb_dslash	Number of ‘//’ tokens	statistical_report	DNS or statistical report flag (0/1/2)
http_in_path	Number of ‘http’ tokens in the path	nb_extCSS	Number of external CSS files
https_token	No-HTTPS flag	login_form	Login form existence indicator (0/1)
ratio_digits_url	Digit ratio in the URL	popup_window	Popup window usage indicator (0/1)
ratio_digits_host	Digit ratio in the hostname	empty_title	Empty title indicator (0/1)
port	Port specified in URL (0/1)	domain_in_title	Whether the domain appears in the page title
tld_in_path	TLD included in path (0/1)	domain_with_copyright	Whether the domain appears near copyright text (0/1)
tld_in_subdomain	TLD included in subdomain (0/1)	label	Phishing label (0/1)
abnormal_subdomain	Abnormal subdomain indicator		

**Table 4:** Reproduction-based and controlled-adaptation comparison with recent state-of-the-art phishing and quishing detection methods on the same decoded-URL phishing dataset.

Model	Acc.	Prec.	Rec.	F1-Score	AUC	F1 Gap	AUC Gap
Haq et al. 1D-CNN [42]	0.9144	0.9266	0.9000	0.9131	0.9733	−0.0202	−0.0019
Phish-Jam Super Learner [43]	0.9001	0.8980	0.9025	0.9002	0.9693	−0.0331	−0.0059
AntiPhishStack Meta-XGB [44]	0.9090	0.9154	0.8990	0.9071	0.9666	−0.0262	−0.0086
Trad and Chehab QR Pixel XGBoost [8]	0.7774	0.7703	0.7887	0.7794	0.8621	−0.1539	−0.1131
Aliyazicioglu URL-Attribute RF [45]	0.8963	0.8920	0.9017	0.8968	0.9604	−0.0365	−0.0148
BERT-PhishFinder DistilBERT [46]	0.9450	0.9648	0.9238	0.9438	0.9844	+0.0105	+0.0092
**WHALE**	**0.9330**	**0.9285**	**0.9382**	**0.9333**	**0.9752**	–	–

*Note:* Boldface in the method column indicates the proposed method, and bold values indicate the best performance for each metric.

**Table 5:** Comparison of different ML models on the same dataset (in %).

Metric	LR	RF	XGB	EBM	SVM	WHALE
Acc.	86.94	91.08	92.77	90.60	89.94	**93.86**
Prec.	87.53	90.41	92.48	90.18	90.91	**93.08**
Recall	86.16	91.91	93.12	91.12	88.77	**94.78**
F1-score	86.84	91.15	92.80	90.65	89.83	**93.92**

*Note:* Bold values indicate the best performance for each metric.

**Table 6:** Dynamic detection performance of WHALE.

Evaluation	Test n	Precision	Recall	Specificity	F1-Score
Independent test	1140	0.895	0.946	0.882	0.920
10-run mean	1134.8	0.895	0.915	0.882	0.904

**Table 7:** On-Device runtime overhead of WHALE.

Stage	Metric	Mean (ms)	Median (ms)	Max (ms)
Static	Feature extraction	55.25	50.30	616.68
	Model input/inference	3.49	3.35	10.55
	Final threshold decision	0.01	0.01	3.19
	Internal subtotal	58.75	53.70	617.69
Dynamic	CRP detection point	1473.56	1145.42	21,948.73
	Decoy probe after CRP	1572.52	1525.27	3442.45
	Submit after probe	535.15	517.57	5008.94
	State59 observation	2049.06	2315.42	4669.12
	HGB inference-only	7.74	1.12	139.91
	Final decision after State59	8.69	2.74	140.20
	Internal subtotal	4165.42	4691.51	10,027.98

**Table 8:** Model-asset-size comparison.

Method	Compared Scope	Size (MB)
PhishHunter-XLD [47]	XGBoost + char-LSTM + DistilBERT	256.718
WHALE	Static model + dynamic HGB	0.681

**Table 9:** Inference-only runtime comparison with PhishHunter-XLD [47].

Method	Model	Mean (ms)	Median (ms)
PhishHunter-XLD [47]	XGBoost inference	0.422	0.408
	char-LSTM inference	1.566	1.497
	DistilBERT inference	116.353	114.022
	Total ensemble inference	118.341	115.953
WHALE	Static TFLite inference	0.024	0.024
	Dynamic HGB inference	0.063	0.056
	Static + dynamic model inference	0.088	0.080

**Table 10:** Static and dynamic detection mobile resource overhead of WHALE.

Stage	Resource	Mean	Median	P95
Static	CPU time (ms)	720.133	531.500	1968.100
	CPU one-core (%)	54.493	46.939	120.666
	CPU all-core (%)	6.812	5.867	15.083
	PSS change (MB)	−0.139	1.534	4.048
	Java heap change (MB)	−0.164	1.259	3.319
	Native heap change (KB)	29.655	35.500	500.100
	Battery change (%)	−0.011	0.000	0.000
	Charge change (mAh)	−0.404	0.000	0.000
Dynamic	CPU time (ms)	1409.766	1142.500	3186.050
	CPU one-core (%)	19.220	17.912	42.765
	CPU all-core (%)	2.403	2.239	5.346
	PSS change (MB)	0.142	0.193	5.271
	Java heap change (MB)	0.174	0.419	5.405
	Native heap change (KB)	162.277	67.000	791.700
	Battery change (%)	−0.017	0.000	0.000
	Charge change (mAh)	−0.875	0.000	0.000

**Table 11:** Integrated mobile resource overhead of WHALE.

Stage	Resource							
	CPU Time	CPU One-Core	CPU All-Core	PSS Change	Java Heap Change	Native Heap Change	Battery Change	Charge Change
	(ms)	(%)	(%)	(MB)	(MB)	(KB)	(%)	(mAh)
Integrated	2129.899	20.411	2.551	0.003	0.010	191.932	−0.027	−1.278

**Table 12:** Adversarial cases considered in WHALE.

Adversarial Case	Attacker Strategy	Runtime Signals Observed by WHALE	Remaining Limitation
Cloaking/interaction-gated exposure	The page initially appears benign. Credential fields or phishing elements are exposed only after user interaction.	Controlled interaction in the Sandbox WebView. Emergence of credential-relevant fields after interaction. Pre/post-submit form and credential-role transitions.	Advanced cloaking may still suppress malicious behavior during sandbox execution. Highly adaptive cloaking remains a limitation.
Delayed redirection	The page delays navigation to avoid immediate redirection-based detection. The malicious transition occurs only after a timer or post-submit wait period.	Time-windowed post-submit observation. Delayed URL, form, or DOM state changes. Delayed credential-flow transition after decoy credential submission.	Very long-delay behavior may exceed the observation window. Extending the observation window increases runtime overhead.
Same-page credential harvesting	The page remains on the same visible URL. Submitted credentials are consumed without a visible domain transition.	Credential-role consumption after decoy credential submission. Credential form removal or reappearance. Hidden-input mutation. Decoy clear behavior. Success-like transition without validation error.	Backend-dead phishing kits without observable submission are excluded from the main binary evaluation. Non-submitting pages require secondary analysis.
JavaScript obfuscation/handler mutation	The page uses script logic or modified handlers to process credential submission. Malicious behavior may be hidden inside event handlers or dynamically generated forms.	Form-action or form-handler mutation. Submit-handler mutation. Hidden-field mutation. Post-submit DOM and credential-role transitions.	WHALE observes the behavioral outcome rather than fully deobfuscating JavaScript. Deep script instrumentation may be required for highly obfuscated logic.
User-agent filtering	The page changes behavior depending on the browser, WebView, device, or user-agent string. The phishing behavior may be exposed only in mobile-like environments.	Execution inside a mobile Sandbox WebView. Runtime state comparison after interaction. Credential-flow behavior observed under WebView execution.	Highly targeted sandbox fingerprinting may still suppress malicious behavior. Broader user-agent diversity is left for future work.
CAPTCHA/MFA-gated phishing	The page hides credential submission behind CAPTCHA, OTP, MFA, or challenge steps. Credential flow may not be observable before the challenge is passed.	CAPTCHA/MFA/challenge indicators. Transition to OTP or secondary credential steps. Hold/retry handling when decoy credential submission is not reliably observed.	Not included in the main strict binary evaluation unless decoy credential submission is observed. Challenge solving is outside the current scope.
Sandbox WebView-aware evasion	The page attempts to detect sandbox execution. Malicious behavior may be suppressed when sandbox execution is suspected.	Session isolation between the Sandbox WebView and User WebView. Decoy-only probing before real credential release. Runtime state comparison under isolated execution.	Complete prevention is not guaranteed. Advanced sandbox fingerprinting remains a future-work limitation.

## Data Availability

The datasets used in this study are openly available. The dataset used for static phishing detection is available at https://doi.org/10.17632/c2gw7fy2j4.3. For dynamic phishing detection, this study used a dynamic credential-flow dataset derived from DynaPD resources. The phishing samples were obtained from DynaPD-based rehosted phishing kits, and the benign samples were derived from benign credential-request page candidates based on Alexa/Tranco-ranked legitimate website resources. The test set was constructed from the same dynamic dataset split and was supplemented with additional benign credential-flow samples collected from live, Tranco-ranked, and crawled legitimate webpages for robustness evaluation. The original DynaPD repository is available at https://github.com/code-philia/DynaPD (accessed on 1 May 2026). This research is limited to cybersecurity and QR-code-based phishing detection, which is beneficial for improving mobile and web security. The authors acknowledge that phishing detection research may have dual-use potential if misused. The study was designed and reported to support defensive security purposes only, and the authors have taken precautions to avoid providing operational instructions for conducting phishing attacks. The authors advocate responsible deployment, ethical considerations, regulatory compliance, and transparent reporting to mitigate misuse risks and foster beneficial outcomes.

## Abbreviations

The following abbreviations are used in this manuscript:

QR	Quick Response
URL	Uniform Resource Locator
HTML	HyperText Markup Language
DOM	Document Object Model
ML	Machine Learning
LR	Logistic Regression
RF	Random Forest
XGB	Extreme Gradient Boosting
HGB	Histogram Gradient Boosting
EBM	Explainable Boosting Machine
SVM	Support Vector Machine
CRP	Credential-Request Page
PSS	Proportional Set Size
WHALE	WebView-Based Hybrid Analysis of Link and Event
TP	True Positive
TN	True Negative
FP	False Positive
FN	False Negative
